# Dual niche modeling with GEE and SHAP for predicting habitat shifts of *Haloxylon ammodendron* and *Cistanche deserticola* under climate change

**DOI:** 10.1371/journal.pone.0338809

**Published:** 2025-12-19

**Authors:** Jing-Xia Guo, Yan Wu, Teng-Da Zhang, Feng-Long Lv, Yi-Fan Gong

**Affiliations:** Baotou Medical College, Baotou, Inner Mongolia, China; University of Uyo, NIGERIA

## Abstract

*Haloxylon ammodendron*, a keystone woody species, and its parasitic plant, *Cistanche deserticola*, play critical roles in sustaining arid ecosystems and supporting regional economies. However, their distribution is increasingly threatened by global climate change. Here, we propose a dual niche modeling framework that integrates climate and soil suitability layers using a multi-model ensemble approach combined with interpretable machine-learning techniques, specifically SHapley Additive exPlanations (SHAP). Using CMIP6 scenarios (SSP126, SSP245, and SSP585), we predicted the current and future potential habitats for both species. The results demonstrated that the ensemble models delivered robust performance, surpassing the accuracy of single-model predictions. Currently, suitable habitats are concentrated in northwestern China as well as parts of Mongolia and Kazakhstan. Under SSP585 (2081–2100), *H. ammodendron* habitats are projected to shrink by 56.2%, whereas *C. deserticola* is expected to lose more than 97% of its habitat, nearly disappearing from Central Asia. Key climatic drivers include temperature seasonality and precipitation patterns, whereas the soil water-holding capacity and gravel content significantly affect local suitability. Niche overlap analysis revealed a strong host dependency for *C. deserticola.* However, the climate–soil niche congruence is projected to decrease under future scenarios, indicating the potential risks of ecological decoupling. This integrative and interpretable approach offers a scalable tool for biodiversity assessment and provides actionable insights for conservation planning in climate-sensitive, arid ecosystems.

## Introduction

Amid the escalating challenges of global warming, the frequency of extreme weather events has increased significantly, posing significant threats to ecosystem stability. This has resulted in a decline in biodiversity at multiple levels and has created substantial challenges for the survival of numerous plant species [1,2]. Since the beginning of the 21st century, the global population has neared 8 billion, with human activities affecting more than 70% of Earth’s land surface [3]. These impacts have intensified the degradation of natural environments, especially in arid ecosystems, where plant habitats are experiencing unprecedented disturbances [4].

Against this backdrop, typical host-parasite plant systems in arid ecosystems, such as *Haloxylon ammodendron–Cistanche deserticola*, serve as ideal models for understanding plant responses to multiple environmental stresses because of their strong ecological interdependence and co-evolutionary mechanisms [5,6].The relationship shows a high degree of host specificity, with *H. ammodendron* acting as the primary and most compatible host that supports a high prevalence of parasitism [7,8]. Moreover, the two partners exhibit contrasting drought-adaptive strategies: the host relies on a deep-rooted C4 photosynthetic pathway [9], whereas the parasite reduces vegetative structures and accumulates osmolytes such as mannitol and betaine to withstand desiccation [10].

The niche theory conceptualizes a species’ habitat requirement as a multidimensional environmental space [11,12], and Ecological Niche Models (ENMs) provide a more robust assessment of species’adaptability to climate change by integrating both climatic and non-climatic factors rather than relying solely on climatic correlations. By coupling species occurrence data with environmental variables, ENMs enable prediction of predict potential distributions and quantify overlapping niche relationships [13,14].This evaluation is essential to uncover evolutionary trends in their co-distribution patterns and provide a scientific foundation for the conservation and restoration of endangered plants in arid regions.

*C. deserticola* Y.C. Ma belongs to the family Orobanchaceae and genus Cistanche Hoffmanns. & Link. It is a perennial parasitic herb known for its exceptional medicinal value, earning it the epithet “desert ginseng.” Its dried fleshy stem has been used in traditional Chinese medicine since ancient times, as documented by Gang Mu (1619), for properties such as kidney tonification, essence replenishment, and bowel relaxation. Modern research has confirmed its diverse health benefits, including its anti-aging effects, memory enhancement, and improved sexual function [15-, 16,17]. Beyond its pharmacological importance, *C. deserticola* is also likely to play notable ecological roles in desert ecosystems. Its presumed roles include interacting with multiple trophic levels by providing floral resources to pollinators and potentially contributing to food webs, although direct evidence for these remains limited. In contrast, its population-level impacts are well-documented: parasitism imposes significant feedback on host survival and vigor, with heavily parasitized *H. ammodendron* individuals showing reduced growth and regeneration [9,18]. Furthermore, evidence suggests that infection intensity is density-dependent, as parasite prevalence increases in dense host patches where root-to-root contact is more frequent [19].

Due to its lack of chlorophyll, *C. deserticola* is entirely dependent on host plants for water and nutrients. Its primary host is *H. ammodendron* (C. A. Mey.) Bunge, a perennial shrub in the family Chenopodiaceae, and genus Haloxylon Bunge. *H. ammodendron* is a quintessential ultra-xerophyte, widely distributed across the arid regions of northwestern China. It exhibits exceptional tolerance to drought, saline-alkaline stress, and wind erosion, making it a key species in arid ecosystems [20]. The survival of *C. deserticola* is highly dependent on its host, *H. ammodendron*, which leads to closely overlapping geographical distributions. However, in recent years, habitat degradation of *H. ammodendron* stands has intensified because of overgrazing, logging, and land-use changes, which directly affect the habitat conditions of this parasitic plant. Concurrently, wild populations of *C. deserticola* have declined significantly due to overharvesting driven by medicinal demand, leading to its listing in Appendix II of the Convention on International Trade in Endangered Species of Wild Fauna and Flora (CITES), 2023 (valid as of 23 February 2023). Documented records indicate that *C. deserticola* mainly occurs in northwestern China (e.g., Xinjiang, Inner Mongolia) and southern Mongolia [21], predominantly inhabiting sandy or gravelly soils within arid desert ecosystems.

Existing studies indicate that host-parasite plant systems are particularly sensitive to environmental changes, with their niches strongly influenced not only by climatic factors but also by microenvironmental conditions such as soil moisture and texture [22]. For example, Lu et al. [23] applied MaxEnt models to predict the potential distributions of four endangered holoparasite–host pairs in China, including *C. deserticola*–*H. ammodendron*, projecting future climate scenarios and revealing significant expansion, contraction, and spatial mismatches in parasite–host distributions. In addition, He et al. [7] modeled the suitable habitats of *C. deserticola* and *H. ammodendron* using MaxEnt, incorporating both climatic and soil variables and analyzing ecological niche overlap. These studies demonstrate the feasibility and transferability of ENMs for predicting range shifts and characterizing ecological suitability in host–parasite systems. Consequently, it is essential to develop highly interpretable ENMs that integrate climatic, edaphic, and host-associated variables to better capture the spatial patterns of ecological suitability for parasitic desert species. This approach facilitates the assessment of the potential impacts of environmental changes on symbiotic stability and future distribution, thereby providing a scientific basis for ecological restoration and species conservation in arid regions.

This study examined the effects of climate change and soil conditions on the ecological niche relationship between the typical host-parasitic plant pair *C. deserticola* and its host *H. ammodendron* in arid ecosystems. Recognizing the complementary roles of climate and soil factors in shaping plant habitats, we used an integrated modeling approach based on the Google Earth Engine (GEE) to systematically evaluate the mechanisms by which multiple environmental factors influence niche overlap [24]. First, using current and future climate data with high spatial resolution, climate niche models were constructed to assess the influence of key variables (e.g., temperature and precipitation) on the potential distribution patterns of *C. deserticola* and *H. ammodendron* under different climate scenarios. This analysis revealed climate-driven dynamic niche shifts in both species. Second, by incorporating constraining soil factors such as texture, water-holding capacity, and soil type, a soil niche model was constructed to characterize the microenvironmental constraints on plant distribution. Subsequently, by applying the minimum suitability rule, the climate and soil niches were integrated to develop a dual-suitability model. This integration improves the prediction accuracy and offers a more comprehensive representation of plant responses to multidimensional environmental gradients in arid ecosystems. Furthermore, to enhance model interpretability, the SHapley Additive exPlanations (SHAP) framework was implemented. SHAP quantifies the marginal contribution of each predictor by borrowing Shapley values from cooperative game theory, ensuring additive and locally accurate explanations of complex model outputs. This approach quantifies the relative contributions of individual environmental variables to model outputs and identifies key factors driving niche differentiation between the parasite and its host species. Finally, niche overlap in the environmental space was quantified using Schoener’s D index, which is a classical metric for measuring niche overlap. This enabled the assessment of host-parasite spatial coupling dynamics and their projected evolution under future climate change, providing critical insights into the co-adaptation and spatial stability of this symbiotic system under multiple environmental constraints.

The primary objectives of this study were (1) to develop an integrated ENM framework for host–parasite plant systems by integrating machine learning with explainable AI, thereby enhancing the accuracy and interpretability of predictions regarding the suitable distribution of *H. ammodendron and C. deserticola*. (2) To quantitatively analyze the differential contributions of environmental factors, including climate, soil, and anthropogenic activities, to the niche spaces of *H. ammodendron* and *C. deserticola,* using the SHAP method. This analysis aimed to elucidate the underlying drivers of the niche overlap patterns. (3) To project the evolution of potential distribution patterns for this host-parasite pair under future climate change scenarios and assess the associated risks posed by shifts in niche overlap on the stability of their symbiotic relationship.This study aimed to provide novel insights into the co-distribution mechanisms of key plant species in arid regions, offering a theoretical foundation and methodological framework for species conservation, ecological restoration, and sustainable resource management.

## Materials and methods

### Occurrence data for *H. ammodendron* and *C. deserticola*

**Fig 1:**
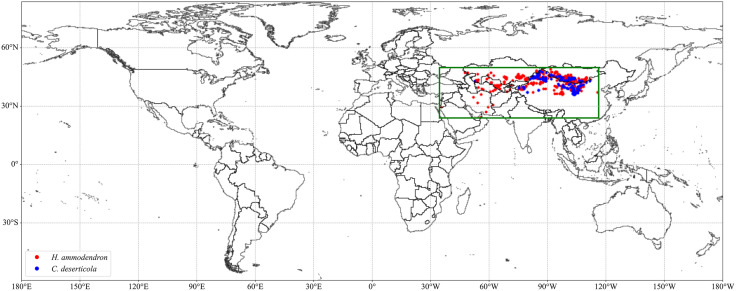
Geographic distribution of occurrence records for *C. deserticola* and *H. ammodendron.* Vector data from USDOS/LSIB_SIMPLE/2017 (public domain) accessed via GEE Data Catalog (https://developers.google.com/earth-engine/datasets/catalog).

Distribution data for *H. ammodendron* and *C. deserticola* were obtained from the following sources: (1) iNaturalist (https://www.inaturalist.org); (2) Global Biodiversity Information Facility (GBIF) [25,26]; (3) National Specimen Information Infrastructure (NSII, http://www.nsii.org.cn/2017/home.php); (4) field survey data; and (5) published scientific literature [27–30]. To reduce spatial sampling bias, occurrence records were first deduplicated at a 1 × 1 km grid resolution using GEE [31]. Specifically, each point was assigned a random value from a 1 km-resolution raster, and duplicates within the same grid cell were removed. This procedure ensures that only one occurrence record is retained per 1 km grid, effectively reducing spatial clustering and minimizing sampling bias for subsequent ecological niche modeling. Ultimately, 199 validated occurrence records for *C. deserticola* and 481 records for *H. ammodendron* (Fig 1) were retained for subsequent ENM analyses.

### Environmental variables

This study initially screened 45 environmental variables that could potentially influence the distribution of *C. deserticola* and *H. ammodendro* (S1 Table in S1 File). These variables included 19 bioclimatic variables from WorldClim 2.1 (January 2020 release; spatial resolution: 1 km) [32]; four topographic variables derived from the Shuttle Radar Topography Mission (SRTM v3) digital elevation model [33]; 20 soil properties extracted from the Harmonized World Soil Database (HWSD v2.0, 1 km resolution, accessed via the GEE Data Catalog; CC BY-NC-SA 3.0) [34]; the Human Footprint Index (HFP) from the Global Human Modification Dataset v3 [35]; and gridded Gross Domestic Product (GDP) data [36]. Future anthropogenic pressure was proxied by population density under Shared Socioeconomic Pathways (SSPs, 1 km resolution, 2020–2100) [37]. The GDP data maintained methodological consistency with the population projections [29,30]. To harmonize the datasets, all layers were reprojected to a common coordinate system (EPSG:4326), resampled to 1 × 1 km resolution, and spatially masked on the GEE platform. Leveraging GEE’s cloud computing capacity enabled efficient processing of these large datasets, reducing temporal artifacts and enhancing the predictive accuracy of the ecological niche models.

To mitigate the effects of multicollinearity on modeling accuracy, a three-stage variable selection protocol was used: (1) Principal Component Analysis (PCA), (2) Pearson’s correlation analysis, and (3) variable importance ranking (S1-S3 Figs in S1 File). This process screened climatic, topographic, and anthropogenic variables, retaining only those that met two criteria: a Pearson correlation coefficient |**r**| < 0.8 and high explanatory power in preliminary models [38]. The final set of 11 variables selected for climate niche modeling comprised four temperature metrics (BIO1, BIO4, BIO8, BIO9), two precipitation metrics (BIO12, BIO15), three topographic factors (elevation, aspect, and slope), and two anthropogenic indicators (GDP and HFP) (S1 Table in S1 File).

### Model construction

This study utilized the GEE platform to develop a high-resolution niche modeling workflow using Python API to independently simulate the ecological suitability distributions of *C. deserticola* and *H. ammodendron*. The modeling process comprises two main steps: (1) delineating the geographic distribution ranges of the species to ensure that model training was confined to their actual ecological habitats, and (2) applying ensemble tree-based models (e.g., Random Forest [RF] and Gradient Boosting [GB]) to assess the impact of current and future environmental variables on the ecological suitability of species and to project their potential distribution patterns under future scenarios.In the study region, SSP1–2.6 is associated with relatively modest increases in extreme heat (>40°C) and drought frequency, whereas SSP5–8.5 projects a sharp rise in both, consistent with IPCC AR6 and regional climate studies [39–41]. These extremes amplify evapotranspiration and soil water deficits, which propagate through the ensemble models as strong negative effects on habitat suitability. As a result, pronounced contractions in potential distribution are particularly evident under the high-emission SSP5–8.5 scenario.

The modeling and analysis were primarily conducted using the GEE Python API (v1.1.2) and Python (v3.12.3), with workflows implemented in Jupyter Notebook (v1.1.1). Data processing and visualization utilized widely used Python libraries, including pandas (v2.2.3), NumPy (v2.0.1), Matplotlib (v3.10.0), and Seaborn (v0.13.2). Additionally, R (v4.2.764) was used for ecological statistical analysis and visualization, leveraging packages such as ade4 (v1.7-22), vegan (v2.6-10), factoextra (v1.0.7), and ggplot2 (v3.5.1). This multi-platform, multi-tool collaborative approach significantly enhances the predictive accuracy and interpretability of the model.

### Training data

In ENM, the quality of the training data is a critical factor that influences the accuracy and reliability of model predictions [42]. Studies have shown that modeling approaches that combine species occurrence records with pseudo-absence data significantly enhance the ability of a model to characterize potential species distribution patterns [2,31,43]. Given the limited sample size of occurrence records for *C. deserticola* and *H. ammodendron*, this study implemented a systematic pseudo-absence data screening framework to improve the rigor and robustness of the model [32].

To address the issue of traditional random sampling potentially misclassifying highly suitable areas as ‘absence’ [44], this study implemented a dual-filtering strategy that integrated environmental profiling with K-means clustering [32]. K-means was selected due to its computational efficiency and scalability to large raster datasets in GEE; however, its sensitivity to k and initial centroids was mitigated by performing multiple runs with random initializations and averaging the results.This approach ensured that pseudo-absence points were sampled exclusively from low-suitability areas or non-representative ecological spaces [45], effectively reducing false-negative errors caused by niche space overlap, while enhancing the discriminative capacity of the model.

Spatial block cross-validation was used to evaluate the robustness of the model against spatial heterogeneity [45–47]. The study area was divided into contiguous spatial blocks, with 70% randomly allocated for training and 30% allocated for validation purposes. This spatial segregation strategy effectively mitigated the biases caused by spatial autocorrelation. The modeling process was repeated over ten iterations, each generating balanced pseudo-absence points equal in number to the presence records within each training and validation block, thereby enhancing predictive stability.

### Construction and evaluation of climate niche models

Given the differential ecological responses and inherent complexities in modeling distinct species, this study developed and implemented an ensemble modeling framework for climate niche models of *C. deserticola* and *H. ammodendron*. The framework integrates four classifiers: two RF models (RF_simple and RF_interm) and two gradient-boosted decision tree models (GBDT_simple and GBDT_interm).The framework integrates four classifiers: two Random Forest models (RF_simple and RF_interm) and two Gradient Boosted Decision Tree models (GBDT_simple and GBDT_interm). The “simple” models correspond to baseline single-algorithm configurations with relatively low model complexity (e.g., smaller minimum leaf population or shallower tree depth), whereas the “interm” models represent intermediate-complexity ensembles that allow deeper tree growth and a larger number of boosting iterations. RF and GBDT were selected because they tolerate non-linear responses, high-order interactions, and missing covariates, all of which are common in heterogeneous desert landscapes. The final suitability predictions were generated by averaging the probability outputs from the four independent models. This ensemble approach enhances the prediction stability and robustness.

Each model underwent ten repeated training and prediction iterations to generate continuous probability surfaces. To enhance interpretability, the optimal classification threshold was determined for each iteration by maximizing the sensitivity and specificity per iteration [48]. The mean optimal threshold was then used to convert continuous probabilities into binary distribution maps (suitable/unsuitable), thereby visualizing potential species distributions.

Model performance during cross-validation was evaluated primarily using the Area Under the Receiver Operating Characteristic Curve (AUC-ROC), supplemented by sensitivity, specificity, True Skill Statistic (TSS), and the Boyce index. This multi-metric approach assesses classification performance, stability, generalization capacity, and predictive robustness across environmental scenarios, thereby ensuring scientifically rigorous niche modeling outcomes.

### Soil niche model construction

The Harmonized World Soil Database version 2.0 (HWSD v2.0) was the primary source of the soil variables. This joint FAO/IIASA (Food and Agriculture Organization of the United Nations/Food and Agriculture Organization of the United Nations) product offers global data at a 1-kilometer resolution on soil morphological, chemical, and physical characteristics, standardized according to the FAO-1990 and WRB (World Reference Base for Soil Resources) classification systems. We used William Ouellette’s processed version in which all string fields were converted into numerically encoded formats arranged alphabetically, thereby facilitating quantitative niche modeling analyses [27].

Soil variables were obtained from HWSD v2.0 and processed using the same three-step selection procedure as applied to the climate variables (S4-S6 Figs in S1 File). Fourteen key edaphic variables were retained to represent major soil properties (e.g., texture, bulk density, drainage, root depth, and water-holding capacity) that govern species distribution (S1 Table in S1 File).

To fully harness the modeling potential of soil variables, an ensemble framework was developed consisting of two RF models (RF_simple and RF_interm) and two GBDT models (GBDT_simple and GBDT_interm). Following the climate niche modeling protocol, all models were run with ten repeated iterations, and their probability outputs were averaged to enhance robustness. As decision-tree-based, non-parametric methods, RF and GBDT automatically capture complex non-linear relationships without assuming normality and handle categorical variables. William Ouellette’s preprocessed data featured numerically encoded categorical variables, allowing direct input into the models without the need for one-hot encoding. This data structure is particularly well-suited for tree-based models, as it effectively prevents dimensionality inflation and improves computational efficiency.

Current studies use two primary strategies to enhance the accuracy of species distribution predictions by integrating soil factors: combined modeling, which incorporates both soil and climate variables into unified models [49], and independent modeling, in which separate soil and climate niche models are constructed and then spatially overlaid to assess joint suitability [50]. Preliminary attempts to develop a combined model using eight climatic and 14 soil variables revealed a limited contribution from soil factors and only marginal performance improvements (S7 Fig in S1 File), indicating inadequate capture of soil influences. Consequently, an independent modeling approach was adopted. Following the “bucket effect” principle, predictions from both niche models were integrated via spatial overlay using the “min” strategy [51].

The operational workflow proceeds sequentially in three key stages. First, climate suitability distribution maps were generated for *H. am*modendron (S8 Fig in S1 File) and C. deserticola (S9 Fig in S1 File), accompanied by the corresponding soil-suitability maps (S10 and S11 Figs in S1 File). Subsequently, the soil suitability map of each species was overlaid onto its climate suitability baseline, and the minimum value per pixel was calculated to derive the dual-factor suitability distribution maps. Finally, to account for the dependency of *C. deserticola* on its host plant (*H. ammodendron*), the host’s suitability map was integrated using the same “min” strategy, such that the final suitability of the parasite is constrained by the least suitable factor among climate, soil, and host availability. This approach ensures the biological realism of the co-suitable maps, as *C. deserticola* can only occupy areas where both environmental conditions and host presence are adequate.

This study used the Maximum Training Sensitivity and Specificity method to establish uniform suitability thresholds [38] for the current period, converting probability maps into binary distribution maps. For future projections (2081–2100), MTS thresholds could not be directly calculated due to the absence of occurrence data; therefore, thresholds derived from the current period were applied. Threshold robustness was further evaluated by conducting a ± 5% sensitivity analysis(S2 Table in S1 File), which showed minimal changes in the spatial extent and pattern of predicted suitable areas. During the suitability overlay analysis, higher thresholds across multiple model outputs were applied to delineate “co-suitable areas.” This dual-threshold approach ensures robust and biologically realistic maps that are comparable across temporal scales and climatic scenarios.

### SHAP analysis

To further elucidate the response mechanisms of environmental variables in species distribution models and their relative contributions to predictions, this study used a SHAP methodology [52,53] for interpretability analysis of ensemble models developed on the GEE platform. SHAP was preferred over traditional importance-based metrics such as the Gini index because it provides signed and locally consistent estimates of each variable’s contribution, enabling the identification of both positive and negative effects on species suitability. Moreover, SHAP captures non-linear and interaction effects that are often overlooked by classical importance measures, thereby enhancing model interpretability in heterogeneous desert environments [54].Based on game theory, SHAP is a model interpretation technique based on the Shapley values. In SHAP, each predictor is treated as a “player” in a cooperative game whose payout corresponds to the model prediction, and the Shapley value fairly distributes this payout among predictors according to their marginal contributions across all possible feature coalitions [55]. This ensures that the contribution of each environmental variable to model predictions is estimated in a consistent and equitable manner, providing both global and local interpretability of species-environment relationships. It quantifies the marginal contribution of individual features to model predictions by considering all possible combinations of feature subsets. This approach enables a unified assessment of the feature importance through both global and local interpretations.

The SHAP value is formally defined as:


$$\phi_{i}(f,x)=\sum_{S\subseteq F\backslash \{i\}}\frac{|S|!\left(|F|-|S|-\text{1}\right)!}{|F|!}\left[f_{S\cup \{i\}}\left(x_{S\cup \{i\}}\right)-f_{S}\left(x_{S}\right)\right]$$

where $\phi_{i}(\text{f},\text{x})$ denotes the contribution of feature to the model output f for sample, F represents the complete feature set, S⊆F\{i} indicates a feature subset excluding i; and ${\text{f}}_{\text{S}}({\text{x}}_{\text{S}}\backslash textrm\mathrm{and}\;{\text{f}}_{\text{S}\cup \{\text{i}\}}\left({\text{x}}_{\text{S}\cup \{\text{i}\}}\right)$ corresponds to the model predictions using feature subsets and S∪{i}, respectively.

Because GEE lacks SHAP computational capabilities, the training data were exported to reconstruct identical RF/GBDT models locally in Python. Using SHAP’s TreeExplainer, we derived per-feature contributions (SHAP values) to approximate the environmental response patterns of the original GEE models.

To enhance the robustness of the interpretation, a SHAP analysis was conducted on four ensemble models comprising dual RF/GBDT configurations. The per-sample SHAP values for the species presence predictions were aggregated through point-wise averaging across the models, resulting in an ensemble SHAP matrix. This matrix simultaneously characterized the variable importance hierarchies and response patterns across the integrated model.

Beyond ranking global variable contributions, this methodology quantifies local-scale effects, including the direction (positive or negative) and intensity of environmental variables across their value spectra, for distribution predictions. SHAP visualizations further delineate the response threshold of species and sensitivity ranges along environmental gradients, providing actionable insights for ecological adaptation research, habitat suitability assessments, and science-based conservation prioritization.

### Niche overlap analysis

Niche overlap refers to a phenomenon in which two or more species share resources or occupy similar ecological niches within an ecosystem [56]. Quantifying this phenomenon is crucial for elucidating the competitive relationships among species, understanding the mechanisms of niche differentiation, and revealing potential patterns of coexistence.

This study used the GEE platform to quantitatively assess the ecological niche overlap between *C. deserticola* and *H. ammodendron* using Schoener’s D index. It analyzed their niche similarity under current climatic conditions, examined trends in niche dynamics across different climate scenarios (current, SSP1–2.6, SSP2–4.5, and SSP5–8.5), and evaluated the coupling relationship and degree of overlap between climatic and edaphic (soil) niches. Schoener’s D index is defined as follows:


$$\text{D}=\text{1}-\frac{\text{1}}{\text{2}}(\sum \left|{\text{p}}_{\text{X},\text{i}}-{\text{p}}_{\text{Y},\text{i}}\right|)$$

where ${\text{p}}_{\text{X},\text{i}}\;\text{and}\;{\text{p}}_{\text{Y},\text{i}}$ denote the habitat suitability index (HSI) for species X and species Y (or for the same species under scenarios X and Y) within the grid cell i. PX or PY represents the HSI values of the same species under different climate scenarios X and Y. Schoener’s D index ranged from 0 to 1. A value of 0 indicated completely separated niches, whereas a value of 1 indicated complete niche identity (overlap). This index quantifies the spatial congruence between predicted distributions by comparing HSI values across grid cells, thereby reflecting the similarity in species distribution patterns [57]. Schoener’s D index overlap is categorized as: very low (0.0 ≤ D < 0.2), low (0.2 ≤ D < 0.4), moderate (0.4 ≤ D < 0.6), high (0.6 ≤ D < 0.8), and very high (0.8 ≤ D ≤ 1.0) [58,59].

## Results

### Niche model evaluation

To systematically evaluate the predictive performance of the niche models, multiple evaluation metrics were used, including AUC-ROC, sensitivity, specificity, TSS, and precision. Additionally, the Boyce index was incorporated to further assess the predictive consistency of climate niche models. Ten iterations were performed on independent datasets for each model. The probability outputs from each iteration were averaged to generate climatic suitability distribution maps for *H. ammodendron* and *C. deserticola* (S8 and S9 Figs in S1 File), and edaphic (soil) suitability distribution maps (S10 and S11 Figs in S1 File). The comprehensive evaluation metrics for all the models are summarized in Tables 1 (climatic models) and 2 (edaphic models). Detailed results for each iteration are provided in Supplementary S3–S6 Tables in S1 File.

**Table 1:** Average performance metrics of climatic niche models.

Species	Model	AUC-ROC	TSS	Boyce Index	Sensitivity	Specificity	Precision
***H. ammodendron***	Ensemble Model	0.9559 ± 0.0089	0.8168 ± 0.0319	0.8575 ± 0.0426	0.9534 ± 0.0240	0.8017 ± 0.0599	0.8534 ± 0.0363
***C. deserticola***		0.9199 ± 0.0298	0.7596 ± 0.0578	0.9143 ± 0.0520	0.9648 ± 0.0288	0.7242 ± 0.0444	0.8460 ± 0.0806

To compare the model performance across different modeling strategies, this study analyzed the efficacy of four base models versus ensemble models in both climate and soil ENM (S7 and S8 Tables in S1 File). As shown in Table 1, the ensemble modeling strategy demonstrated superior or more consistent performance across most climate niche modeling metrics. For *H. ammodendron*, the ensemble model achieved near-optimal or highest values for key evaluation metrics, including AUC-ROC (0.9559 ± 0.0089), TSS (0.8168 ± 0.0319), and precision (0.8534 ± 0.0363), indicating a robust performance. Furthermore, it maintained a high sensitivity (0.9534 ± 0.0240) and specificity (0.8017 ± 0.0599), demonstrating a balanced performance in identifying suitable habitats while effectively minimizing false positives.

For *C. deserticola*, the ensemble model also demonstrated strong performance across key metrics, including AUC-ROC (0.9199 ± 0.0298), TSS (0.7596 ± 0.0578), and the Boyce index (0.9143 ± 0.0520), indicating a robust predictive power and generalization capacity. With a sensitivity of 0.9648 ± 0.0288, the model exhibited exceptional capability for identifying potentially suitable habitats. Overall, the ensemble approach consistently outperformed the individual models in climate niche modeling for both species and was therefore selected as the final predictive tool to ensure reliable and broadly applicable results.

The predictive performance of the various models varied within the soil niche (Table 2). Overall, the ensemble models demonstrated superior capability for modeling *H. ammodendron* and *C. deserticola*. For *H. ammodendron*, the ensemble model delivered exceptional performance, achieving an AUC-ROC of 0.9704 ± 0.0093 and TSS of 0.9027 ± 0.0287, approaching or exceeding the best results observed among the individual models. Furthermore, it attained well-balanced sensitivity (0.9410 ± 0.0242) and specificity (0.9411 ± 0.0292), along with a high precision of 0.8942 ± 0.0385. This robust combination of metrics further validated the reliability and predictive accuracy of the model. Although the overall metrics for *C. deserticola* were slightly lower than those for *H. ammodendron*, the ensemble model still exhibited a strong predictive capability, achieving an AUC-ROC of 0.9158 ± 0.0290 and TSS of 0.7026 ± 0.0707. Its sensitivity (0.9412 ± 0.0807) indicated strong detection ability. Although the specificity (0.7096 ± 0.0865) and precision (0.7467 ± 0.0628) were moderately reduced, they remained superior to those of several individual models, underscoring the stability and practical potential of the ensemble approach. In summary, ensemble models consistently demonstrated high performance in both climate and soil niche modeling, particularly in terms of enhancing model stability and overall predictive accuracy. Consequently, the ensemble modeling approach was selected as the definitive niche modeling framework in this study to improve the scientific rigor and ecological relevance of our findings.

**Table 2:** Average performance metrics of soil niche models.

Species	Model	AUC-ROC	TSS	Sensitivity	Specificity	Precision
***H. ammodendron***	Ensemble Model	0.9704 ± 0.0093	0.9027 ± 0.0287	0.9410 ± 0.0242	0.9411 ± 0.0292	0.8942 ± 0.0385
***C. deserticola***		0.9158 ± 0.0290	0.7026 ± 0.0707	0.9412 ± 0.0807	0.7096 ± 0.0865	0.7467 ± 0.0628

### Key predictors

#### Interpretation of key factors via model variable importance.

Niche modeling predictions indicated that the key environmental variables influencing the potential geographic distribution of *C. deserticola* and *H. ammodendron* under climate change scenarios included bio1, bio4, bio8, bio9, bio12, HFP, and elevation (S3 Fig in S1 File). Importantly, the contribution rate of each key climate variable exceeded 4%, demonstrating that temperature and precipitation were the primary climatic drivers of the distribution patterns of both species in the study area. Notably, HFP exhibited contribution rates between 3% and 5% for both species, suggesting a significant influence of human activity on the natural distribution patterns of these species. Furthermore, elevation showed contribution rates of 6.9% for *C. deserticola* and 2.7% for *H. ammodendron*, indicating that topographic factors may modulate species distribution, particularly at the local scale.

Among the soil factors, the key variables influencing the distribution of both species included HWSD_ID, available water capacity of soil (AWC), SHARE, and TEXTURE_USDA, each contributing more than 5%. HWSD_ID emerged as the most explanatory soil variable, accounting for more than 22% of the variation in the distribution models of both *C. deserticola* and *H. ammodendron*, a value that was considerably higher than that of the other variables. This finding demonstrates that the soil type plays a predominant role in determining species distribution patterns. AWC also exhibited a significant influence, contributing nearly 15% to the *H. ammodendron* model and more than 7% to the *C. deserticola* model, underscoring the critical importance of soil water availability for plant survival. Furthermore, SHARE contributed more than 15% to the *C. deserticola* model and exerted a significant effect on *H. ammodendron*, indicating that soil texture is a key regulator of habitat suitability. Variables, such as TEXTURE_USDA and Root_Depth, demonstrated a moderate influence on the models for both species, further underscoring the fundamental role of soil physicochemical characteristics in ecological niche formation. Collectively, soil factors, particularly those related to soil type and texture, played a crucial role in predicting the potential future distribution of these species. Their significant influence is likely to be closely associated with the specific ecological adaptation strategies used by each species.

#### Interpretation of key factors based on SHAP values.

##### Interpretation of key environmental factors using SHAP values:

To further elucidate the contribution and underlying mechanisms of each environmental variable in predicting the potential distribution of species, the SHAP analysis was applied to interpret the output of the reconstructed ensemble model (Figs 3 and 4). Beyond merely ranking variable importance, the SHAP analysis revealed the directional effects (positive or negative) of different value ranges on the predicted probability of species presence. This approach overcomes a key limitation of traditional variable contribution methods, which quantify the overall influence but fail to clarify causal pathways or the directionality of effects.

**Fig 2:**
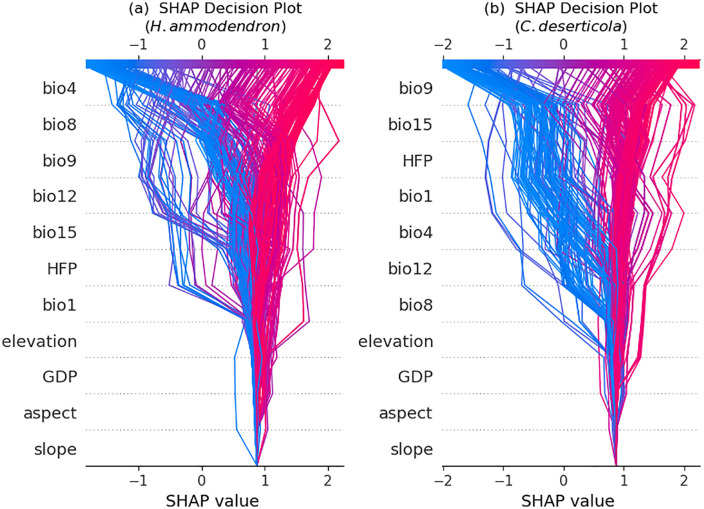
SHAP decision plots showing contributions of environmental variables to prediction pathways for *H. ammodendron* and *C. deserticola.*

**Fig 3:**
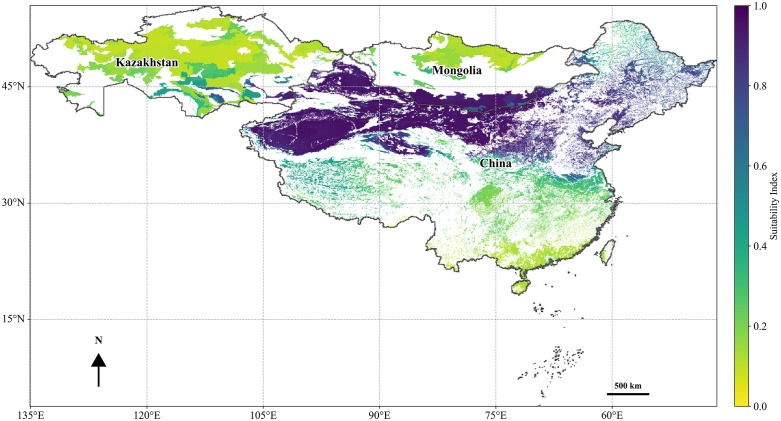
Habitat suitability map for *H. ammodendron.* Vector data were obtained from publicly available datasets in the GEE Data Catalog (https://developers.google.com/earth-engine/datasets/catalog).

**Fig 4:**
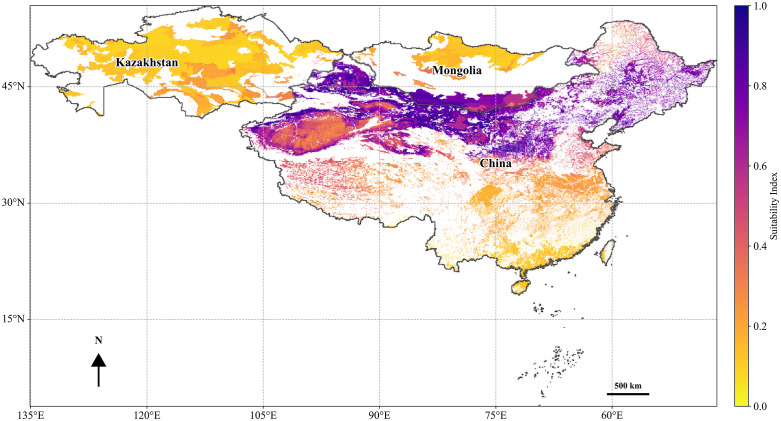
Habitat suitability map for *C. deserticola.* Vector data were obtained from publicly available datasets in the GEE Data Catalog (https://developers.google.com/earth-engine/datasets/catalog).

For *H. ammodendro*n (S12a, S13 Figs in S1 File), bio4 and bio8 emerged as the most influential climatic variables determining their potential distribution, with the SHAP mean absolute values significantly exceeding those of the other factors. The analysis revealed that lower values of bio4 were positively correlated with the predicted probability of presence. Moderate to high values of bio1 were also positively associated with habitat suitability. Furthermore, moderate to high values of bio8 had a significant positive effect on the probability of species occurrence. These relationships suggest that *H. ammodendron* thrives under warmer conditions during the wettest season (bio8), which is potentially associated with the synchronization of thermal and hydrological resources. In other words, warm temperatures coinciding with periods of adequate moisture enhance physiological processes and biomass accumulation. Additionally, the HFP exhibited positive SHAP values within specific ranges, indicating that moderate anthropogenic activities may have facilitated the distribution of *H. ammodendron*. This could stem from the indirect benefits provided by infrastructure, such as water management systems or ecological restoration initiatives. Conversely, elevation exerted a consistently negative influence, reflecting the limiting effects of lower temperatures and moisture deficits at higher altitudes, which significantly reduced the likelihood of species occurrence in these areas.

In *C. deserticola* (S12b, S14 Figs in S1 File), bio9 exhibited the highest SHAP value among the climatic variables, establishing it as the most critical climatic factor influencing potential distribution. Bio9 had a significant positive effect on the predicted probability of presence, particularly within its moderate-to-high range, indicating warmer conditions during the driest quarter of the year. This strongly suggests that *C. deserticola* prefers environments with warmer winters or dry seasons, conditions potentially conducive to the growth of its host, *H. ammodendron*, and initiation of the parasitic process. In contrast, bio15 exhibited a nonlinear relationship with the suitability. The SHAP values peaked under moderate precipitation seasonality, indicating a preference for regions with relatively balanced intra-annual precipitation distribution. Conversely, excessively high seasonal precipitation variation likely constrains growth, particularly by disrupting the critical synchronization of germination and host plant availability. Furthermore, the HFP also showed a positive contribution, especially within the low-to-moderate disturbance range. This finding implies that the potential distribution of *C. deserticola* may benefit from microhabitat amelioration associated with certain anthropogenic activities such as water resource management and desert greening initiatives. Collectively, the SHAP analysis revealed significant differences in the relative importance rankings of key environmental factors, their specific modes of action, and their effect magnitudes. These insights provide a robust theoretical basis for further elucidation of the divergent ecological adaptation strategies and distribution mechanisms of these two species.

Further analysis of the SHAP decision plots (Fig 2) elucidated the cumulative contributions of environmental variables across individual samples, thereby clarifying the mechanistic basis of the prediction results. For *H. ammodendron*, consistent contribution patterns were observed for bio4, bio8, bio9, bio12, and bio15. Most samples exhibited positive SHAP values for these variables, indicating a broadly beneficial and relatively uniform influence of these climatic factors on increasing the predicted presence probabilities with minimal inter-sample variation in their effects. Conversely, the decision paths for *C. deserticola* revealed a considerably greater variability. Marked fluctuations in SHAP values were evident for bio9, bio15, HFP, and bio1, reflecting the heightened sensitivity of this species to localized environmental conditions. Consequently, its distribution pattern was substantially influenced by fine-scale climatic heterogeneity and human activity. This distinctive pattern is likely attributable to its parasitic life history, whereby dependence on host (*H. ammodendron*) distribution and microenvironment results in a more heterogeneous niche.

##### Interpretation of key soil factors using SHAP values:

To further clarify the marginal contributions of soil factors to the potential distribution modeling of the two desert plant species, SHAP analysis was used to interpret the primary soil variables (S18 Fig in S1 File). For H. ammodendron (S15a, S16 Figs in S1 File), the most influential soil variables were CONGRAVEL, SHARE, and AWC. Both CONGRAVEL and SHARE exhibited strong positive effects, indicating a higher probability of habitat suitability in areas with a greater gravel content and elevated organic matter levels. This pattern aligns well with the known adaptations of *H. ammodendron* to arid, well-drained soils and its dependence on soil organic matter. The AWC, which reflects the soil water retention capacity, demonstrated a distinct pattern, with its contribution peaking at moderate-to-high levels. This suggests that both excessively high and low water-holding capacities are suboptimal for the habitat suitability of this species.

In *C. deserticol*a (S15b, S17 Figs in S1 File), the results indicate that SHARE makes the most significant positive contribution to the model predictions. Higher SHARE values suggest that the dominance of specific soil units within a grid cell strongly enhances habitat suitability, implying that *C. deserticola* tends to favor areas in which its preferred soil type occupies a larger proportion of the area. This likely reflects the ecological requirements of both species and their host, *H. ammodendron*. Concurrently, WRB2_CODE demonstrated the highest overall contribution among the variables, signifying a pronounced ecological preference for specific soil types, potentially reflecting a dependency on associated physicochemical properties or microhabitat conditions. In contrast, TEXTURE_USDA exhibited an overall negative trend in SHAP values. The predicted suitability significantly decreased at the extremes of the texture spectrum (e.g., overly compacted or excessively loose soils), suggesting that suboptimal soil textures impose significant constraints on parasitic establishment and subsequent growth. Furthermore, HWSD2_ID (soil type identifier) exhibited notable explanatory power, providing additional evidence that *C. deserticola* has a spatially aggregated distribution within specific soil-type regions. This distribution pattern likely reflects a substantial overlap with the core soil niche occupied by the host plant, *H. ammodendron*.

### Projected changes in potential distribution of *H. ammodendron* and *C. deserticola* under climate change scenarios

The potential distribution of dual-suitability habitats for *H. ammodendron* under the current climatic conditions, based on predictions from integrated climatic and soil niche models, is shown in Fig 3. Its suitable range is primarily located within China, Mongolia, and Kazakhstan, exhibiting a distinct distribution pattern consistent with arid desert ecological zones. Within China, the core suitable areas were concentrated in arid and semi-arid regions, including Inner Mongolia, Xinjiang, Qinghai, Gansu, and Ningxia (Fig 3). In Mongolia, the main distribution occurs in the southern Gobi Desert region, encompassing the South Gobi (Ömnögovi), East Gobi (Dornogovi), and Gobi Altai highlands. In Kazakhstan, suitable habitats were concentrated within the arid zone south of Lake Balkhash, which is closely aligned with the Central Asian desert ecological belt.

The potential habitat distribution of *C. deserticola* under the current climatic conditions is illustrated in Fig 4. Its range is predominantly confined to China and Mongolia and is characterized by a distinct obligate parasitic strategy and adaptation to arid zones. Within China, suitable habitats are concentrated in the desert and semi-desert regions of Inner Mongolia, Xinjiang, and Gansu (Fig 4). In Mongolia, their occurrence is primarily localized to the southern Gobi (Ömnögovi) and parts of the Gobi-Altai region. This distribution closely corresponds to the range of its host plant, *H. ammodendron*, indicating that its parasitic habitat is fundamentally constrained by the ecological niche of the host. Kazakhstan had limited or negligible areas of predicted habitat suitability.

Under future climate scenarios (SSP126, SSP245, and SSP585; 2081–2100), the potentially suitable habitat areas for both *H. ammodendron* and *C. deserticola* are projected to contract significantly (Table 3). Compared with the current climatic conditions, their distribution patterns exhibited a pronounced spatial constriction.

**Table 3:** Projected suitable habitat areas for *H. ammodendron* and *C. deserticola* under current and future climate scenarios (×10³ km²).

Species	Scenario	World	China	Mongolia	Kazakhstan
***H. ammodendron***	current	2456.6786	2098.5570	212.4417	30.2895
	SSP126 (2081–2100)	1045.6188	1009.5175	52.9215	8.5506
	SSP245 (2081–2100)	1180.3887	979.0510	45.4660	10.6074
	SSP585 (2081–2100)	1045.6188	918.8872	33.1057	7.5180
***C. deserticola***	Current	1258.0116	1077.0710	123.2656	0.5189
	SSP126 (2081–2100)	35.7865	35.7865	–	–
	SSP245 (2081–2100)	25.7768	25.7700	–	–
	SSP585 (2081–2100)	24.0196	24.0190	–	–

Global Scale (*H. ammodendron*): The suitable habitat area is projected to decline from approximately 2.4567 million kilometer ² (current) to approximately 1.0456 million kilometer ² under both SSP126 and SSP585, representing a reduction of approximately 57.4%. Under SSP245, the suitable area is slightly higher (approximately 1.1804 million kilometer ²) but still constitutes a substantial loss of approximately 51.9%.

China Scale (*H. ammodendron*): The suitable area contracts significantly across all scenarios, with the most severe reduction under SSP585 (only approximately 0.9189 million kilometer ² remaining; an approximately 56.2% decrease relative to the current value). These projections indicate that extreme future climate conditions will substantially reduce the ecological niche suitability of *H. ammodendron*, exacerbating habitat fragmentation risks, particularly under medium-to high-emission scenarios (SSP245 and SSP585).

In contrast, *C. deserticola* exhibits disproportionately high vulnerability to climate change. Its suitable habitat area is projected to decline dramatically from approximately 1.258 million kilometer ² to approximately 0.0358 million kilometer ² (SSP126), 0.0258 million kilometer ² (SSP245), and 0.0240 million kilometer ² (SSP585), representing losses of more than 97% across all scenarios. In China, this trend mirrors the global patterns. Critically, suitable habitats are projected to be eliminated from Mongolia and Kazakhstan in all future scenarios, indicating catastrophic range contraction and imminent habitat loss. Collectively, climate change poses an extreme threat to the potential distribution of both *H. ammodendron* and *C. deserticola*. The magnitude of habitat loss is particularly severe in *C. deserticola*, jeopardizing the persistence of both species and the essential ecosystem functions associated with their parasitic relationships. These findings underscore the urgent need to develop differentiated conservation strategies for arid ecosystems, prioritizing climate-vulnerable parasitic plants and their obligate host systems in conservation efforts.

### Ecological niche overlap analysis

The degree of niche overlap between *H. ammodendron* and *C. deserticola* exhibited distinct patterns across climate scenarios (Table 4), as quantified by Schoener’s D index (ranging from 0 to 1, where values approaching 1 indicate high niche overlap). For *H. ammodendron*, the overlap between climatic and soil suitability under the current conditions was high (D = 0.8977), demonstrating a strong concordance between these environmental factors in shaping its potential distribution. However, under future climate change scenarios (SSP126, SSP245, and SSP585), this overlap declined slightly, ranging from 0.8564 to 0.8579. This suggests that climatic shifts may reduce the spatial congruence between climate and soil conditions, thereby increasing uncertainty in habitat suitability predictions. For *C. deserticola* (incorporating host suitability), the niche overlap with its host (*H. ammodendron*) was 0.9131 under current conditions. Notably, this overlap was increased slightly under the future scenarios, ranging from 0.9410 to 0.9422. This indicates that the potential habitat of *C. deserticola* remains strongly dependent on host distribution, with an obligate parasitic relationship maintaining high spatial fidelity despite climate change. In summary, *C. deserticola* and *H. ammodendron* will maintain an exceptionally high niche overlap in the future, implying that their obligate parasitic dependence is resilient to projected warming. However, both species exhibited reduced internal concordance between climate and soil suitability. This suggests that climate-driven divergence in key environmental drivers may increase the instability of potential distributions, thereby posing challenges to the recovery and stability of desert ecosystems.

**Table 4:** Schoener’s D Niche overlap between *H. ammodendron* and *C. deserticola.*

Schoener’s D			
Scenario	*H. ammodendron* cliamte-soil	C. *deserticola* cliamte-*H. ammodendron*	*C. deserticola* cliamte-soil
**current**	0.8977	0.9131	0.9016
**SSP126 (2081–2100)**	0.8564	0.9410	0.8868
**SSP245 (2081–2100)**	0.8579	0.9412	0.8861
**SSP585 (2081–2100)**	0.8573	0.9422	0.8853

## Discussion

### Model rationale

To ensure the stability and reliability of the predictions and mitigate stochastic fluctuations caused by the initial model parameter settings or sample partitioning, this study used a strategy of conducting ten iterative runs for each model on an independent test dataset. The prediction probabilities from each iteration were averaged to provide a robust foundation for constructing ENMs for *H. ammodendron* and *C. deserticola*.

During the initial model development phase, an approach integrating climate and soil variables into a single unified ENM was explored to comprehensively capture the responses of species to multidimensional environmental drivers. However, the results indicated a notably low contribution of soil variables within this integrated model (S7 Fig in S1 File). This limitation likely stems from two primary factors: (1) potential collinearity between soil and climate variables, which may have diminished the independent explanatory power of soil factors and obscured their true influence on species distribution, [60] and (2) the common treatment of soil properties as temporally static factors, which, although often reasonable for current conditions, may impair model transferability under future climate scenarios where this assumption of stability could be violated [61]. This static soil assumption may lead to underestimation of the ecological constraints imposed by edaphic factors and, consequently, could affect the interpretation of species’ realized niches. Therefore, conservation recommendations derived from the model should be applied cautiously, acknowledging that shifts in soil conditions under future climate change may alter habitat suitability patterns.

To address these limitations, a separate model construction strategy followed by post-hoc integration was adopted. Distinct climate and soil suitability models were developed for *H. ammodendron*. Reflecting the ecological principle that a suitable habitat requires the simultaneous fulfillment of both climatic and edaphic conditions (the “barrel effect”) [39], the final habitat suitability for each grid cell was determined by taking the minimum value from the respective climate and soil suitability layers. This approach effectively minimizes the impact of variable redundancy and potential biases. Subsequently, the overall suitability of *C. deserticola* within each grid cell was derived by integrating its predicted climate suitability with the soil suitability layer generated for its host (*H. ammodendron*) using the same minimum value method as above. Furthermore, the ensemble models demonstrated a strong performance across multiple validation metrics (AUC-ROC, TSS, sensitivity, and precision) in the independent modeling phase. Notably, predictions for *H. ammodendron* showed high stability and explanatory power. The consistent results across all ten iterations (S3–S6 Tables in S1 File) further substantiate the reproducibility and robustness of the models, confirming the rationale and feasibility of the methodological framework used.

### Importance of key influencing factors

Climate and soil factors play crucial roles in determining the potential distribution patterns of *H. ammodendron* and *C. deserticola*; however, their relative influences and underlying mechanisms show noticeable differences between the two species.

Within climate niche models, temperature- and precipitation-related variables (e.g., bio1, bio4, bio8, bio9, and bio12) consistently demonstrated significant contributions of more than 4% each(S3 Fig in S1 File). This underscores their critical role as limiting resource factors in species distribution. For instance, bio1 and bio12 directly govern plant energy acquisition and water balance, which are classic indicators of climate suitability. These findings align with previous research, highlighting the high sensitivity of arid-zone plants to thermal and moisture regimes [62], suggesting that future climatic shifts may significantly reshape their potential distribution ranges on a broad scale.

Beyond the climatic variables, HFP contributed between 3% and 5% of the models. Although this is lower than the contribution of the primary climate predictors, it indicates the potential influence of anthropogenic activities on ecological patterns. Activities such as land use change, pastoralism, and infrastructure development may indirectly affect species distribution dynamics by disrupting habitat connectivity and degrading habitat quality.

Furthermore, elevation emerged as a highly influential factor for *C. deserticola*, accounting for 6.9% of the explanatory power of the model. This underscores the significant role of local topography in shaping microclimate, soil formation, and vegetation succession, thereby influencing the spatial distribution of this parasitic plant [19].

In the soil niche models, the soil type variable (HWSD_ID) was the most influential, contributing more than 22% (S9 Fig in S1 File), which was significantly higher than that of the other factors. This indicates a strong constraint imposed by soil type on species distribution, which is likely mediated by its effects on root development, nutrient availability, and microhabitat stability [63]. For *H. ammodendron*, AWC contributed nearly 15%, reflecting the critical role of soil water retention in the survival of woody plants in arid regions. In contrast, coarse fragment content (SHARE) contributed more than 15% to the *C. deserticola* model, highlighting its greater dependence on the enhanced water infiltration and host root attachment conditions provided by gravelly soils [64]. Although not dominant, other factors, such as USDA soil texture (TEXTURE_USDA) and effective root_depth (ROOT_DEPTH), exhibited a moderate influence, suggesting that soil physicochemical properties profoundly shape niche formation by regulating plant water acquisition, nutrient uptake, and physiological tolerance.

In summary, the different responses of species to climatic and edaphic factors reflect their distinct ecological adaptation strategies. *H. ammodendron*, a deep-rooted woody sand-fixing plant, exhibits a distribution primarily constrained by water availability and soil water-holding capacity. In contrast, *C. deserticola*, a root-parasitic medicinal plant, is more sensitive to soil texture and microhabitat conditions surrounding the host root system. Consequently, the dual-dimensional (“climate-soil”) independent modeling approach used in this study, followed by integration tailored to the ecology of each species, enhances the plausibility of predictions and aligns more closely with the mechanistic logic of the underlying ecological processes.

### Ecological interpretation of key environmental factors using SHAP values

By leveraging interpretability analysis based on SHAP, this study further elucidated the direction and strength of the effects of climatic and soil variables on the potential distribution of *H. ammodendron* and *C. deserticola*, clearly delineating their divergent environmental adaptation strategies.

Within the climatic dimension, SHAP analysis revealed that *H. ammodendron* exhibited the highest sensitivity to bio4 and bio8, with SHAP values significantly exceeding those of the other climate variables (S12a Fig in S1 File). This indicates greater adaptation to environments characterized by substantial temperature fluctuations and high summer temperatures, consistent with its ecological traits as a drought-tolerant, deep-rooted desert shrub [65]. In contrast, the climatic response of *C. deserticola* was predominantly influenced by bio9, whose SHAP value was markedly higher than those of the other variables (S12b Fig in S1 File). This suggests a stronger dependence of its distribution on specific thermal regimes. This difference likely arises from its parasitic life history, which entails a high dependence on the physiological state of its host (*H. ammodendron*), indicating that the physiological adaptability of the host under extreme climatic conditions indirectly affects the survival prospects of *C. deserticola.*

In the soil dimension, both species exhibited strong responses to key soil attributes, although potentially through different mechanisms. For *H. ammodendr*on (S15a Fig in S1 File), CONGRAVEL, SHARE, and AWC were the most influential factors. This highlights its adaptation to well-drained, stable soils with moderate water retention. In contrast, C. deserticola exhibited the strongest response to SHARE (S15b Fig), demonstrating a pronounced positive relationship. This indicates its reliance on favorable soil aeration and water infiltration conditions provided by gravelly substrates [39]. Furthermore, the soil type variables (WRB2_CODE and HWSD2_ID) also exerted significant explanatory power over its potential distribution, implying that the niche structure of *C. deserticola* is largely constrained by the specific soil environment preferred by its host.

Collectively, the SHAP results not only quantified the importance of environmental variables but also revealed differences in the ecological functions and niche construction mechanisms between the two species. The potential distribution of *H. ammodendron* was co-regulated by climatic and soil factors, with macroclimatic drivers playing a dominant role. In contrast, *C. deserticola* exhibits characteristics typical of a “nested niche”, a situation in which the fundamental niche of the parasite falls entirely within the realized niche of its host, imposing an obligate dependency [66]. Its distribution is highly dependent on the host and influenced by the combined effects of climate, soil, and host factors. This finding underscores the critical importance of incorporating host constraints into ENMs and has significant implications for conservation strategies targeting parasitic plants in arid regions.

### Analysis of potential habitat distribution

Based on a dual-dimensional ENM framework, this study systematically assessed the potentially suitable habitat distribution of *H. ammodendron* and *C. deserticola* under current and future climate scenarios.

The results indicate that, under current climatic conditions, the highly suitable areas for both species are predominantly concentrated in the arid regions of Northwest China and adjacent areas. This core distribution includes the provinces of Inner Mongolia, Xinjiang, Gansu, Qinghai, and Ningxia, which extend into the southern Gobi Desert of Mongolia and parts of the arid zone of Kazakhstan. This spatial pattern closely aligned with the typical desert ecological adaptations of both species, confirming the predictive reliability of the model under baseline conditions.

Under future climate change scenarios (SSP126, SSP245, and SSP585), particularly during the period 2081–2100, potentially suitable habitats for both species are projected to contract significantly. *H. ammodendron* is expected to experience a reduction of more than 50% in its globally suitable area. Specifically, under the high-emission SSP585 scenario, the suitable area decreased to approximately 918,887 km² (918.8872 × 10³ km²), representing a 56.2% decline compared with the current conditions. The response of *C. deserticola* to climate change is even more pronounced, with its suitable habitat projected to shrink globally by more than 97%. Under the SSP585 scenario, its distribution in Mongolia and Kazakhstan was nearly eliminated, indicating a severe trend of range contraction and habitat fragmentation.

This marked contraction in habitat suitability highlights the high climatic sensitivity of both species and underscores the significant challenges faced by endemic arid-land vegetation under global warming. *C. deserticola*, in particular, experiences extreme habitat compression. As an obligate parasitic plant dependent on *H. ammodendron*, this decline is likely due not only to decreasing climatic suitability but also to reduced host availability and potential disruption of their ecological symbiosis. These findings emphasize the importance of incorporating species interdependencies to predict future distribution patterns.

Regional-scale analysis indicates that Northwest China is projected to remain a core climatic refuge for both species in the future. However, both the area and the connectivity of suitable habitat patches within this region are expected to decline significantly. This degradation is likely to result in population fragmentation and the loss of genetic diversity, necessitating increased conservation efforts. Conservation strategies should prioritize this relatively stable zone and focus on maintaining habitat connectivity, implementing long-term ecological monitoring, and adopting climate-adaptive management practices. In conclusion, future climate change is projected to cause substantial range contractions for both *H. ammodendron* and *C. deserticola*, with *C. deserticola* facing an exceptionally high risk of near-total habitat loss. These findings underscore the urgent need to develop targeted strategies for species conservation, desert ecosystem restoration, and climate resilience planning in arid regions.

### Ecological niche overlap analysis

This study quantitatively assessed niche overlap between *H. ammodendron* and *C. deserticola* under various climate scenarios using Schoener’s D index. This analysis aimed to elucidate the influence of combined climate-soil suitability and parasitic dependence on future spatial distribution patterns. The results demonstrated significant differences in the spatial congruence of the ecological suitability between the two species.

For *H. ammodendron*, under current climatic conditions, the niche overlap value between climatic suitability and soil suitability was significantly high (D = 0.8977), indicating a strong synergistic effect of these ecological factors in shaping its distribution. However, under future climate change scenarios, this overlap value decreased (D = 0.8564–0.8579), reflecting reduced spatial congruence between climate and soil suitability. This trend likely results from a mismatch between the spatial shift of climatically suitable areas and the relatively static nature of soil properties—a phenomenon termed “climate-soil decoupling” [45], which leads to increasingly fragmented patterns of its overall suitable habitat.

*C. deserticola* exhibited a stronger dependence on its host in its niche overlap dynamics. When accounting for its parasitic relationship with *H. ammodendron*, the overlap value was 0.9131 under current conditions and increased slightly to 0.9410–0.9422 under future scenarios. This indicates that, even under climate change pressure, the potentially suitable areas of *C. deserticola* remain highly dependent on and closely track the host’s distribution, reinforcing the central role of its parasitic strategy in spatial niche construction. Conversely, when evaluating its intrinsic climate and soil suitability independently (excluding host dependence), the overlap value decreased from 0.9016 to 0.8853–0.8868, which is consistent with the trend observed for the host. This further suggests that asynchronous future changes in environmental factors may weaken the internal consistency of the ecological niche, thereby increasing its susceptibility to habitat fragmentation [67].

Despite these shifts, *C. deserticola* and *H. ammodendron* are projected to maintain a high degree of ecological spatial overlap in the future, thereby demonstrating the relative stability of parasitic ecological strategies in response to climate change. Nevertheless, the risks of habitat quality degradation and loss of habitat integrity resulting from climate-soil decoupling imply that future conservation strategies must simultaneously address host distribution continuity and soil habitat stability to mitigate local extinction risks for parasitic plants under these dual pressures [68].The contrasting vulnerabilities of *H. ammodendron* and *C. deserticola* highlight the need for differentiated conservation strategies. While *H. ammodendron* is sensitive to soil degradation and climatic extremes, *C. deserticola* is additionally constrained by host availability due to its obligate parasitic lifestyle. This interdependence implies that habitat degradation affecting the host may disproportionately impact the parasite, potentially altering community interactions and desert ecosystem functioning. Conservation management should therefore prioritize maintaining host population integrity, preserving suitable soil habitats, and ensuring connectivity between host patches to support the persistence of both species. Targeted restoration of host shrubs in degraded areas may indirectly benefit parasite populations, underscoring the importance of integrated, host-parasite-focused strategies.

### Limitations and future prospects

Despite using multi-model ensemble techniques and ENM strategies to enhance the prediction robustness and accuracy, this study acknowledges several significant limitations that warrant attention in future research. First, uncertainties associated with climate projections remain inherent. Although representative GCMs under the CMIP6 framework were employed, inter-model variability among climate simulations may influence future habitat suitability patterns. Future studies could incorporate ensemble averaging across multiple GCMs to further quantify and reduce climate-related uncertainty.

Second, algorithmic and threshold-selection uncertainties may also affect predictive reliability. While ensemble learning and cross-validation were used to mitigate model dependence, differences among algorithms and the choice of suitability thresholds can introduce additional variability. Sensitivity analyses and model comparison frameworks are recommended for future applications to improve transparency and reproducibility.

Third, this approach does not completely capture climate-soil interactions. Although the independent modeling of climatic and edaphic niches effectively mitigates multicollinearity-induced model instability, it may oversimplify real-world ecological processes. Climate fundamentally shapes soil attributes through its influence on pedogenesis, hydrological cycling, and nutrient fluxes, whereas soil conditions reciprocally modulate the sensitivity of plants to climatic drivers [69]. Decoupling these dimensions may lead to a partial underestimation or bias in characterizing the ecological adaptation mechanisms of species.

Fourth, constraints exist in the dual-model integration methodology. The minimum-value overlay approach (min) used to integrate the climate and soil suitability layers represents an empirical rule-based solution. However, this method inherently fails to account for the potential non-linear synergistic effects between climate and soil factors. Future research should investigate more ecologically grounded integration strategies, such as Bayesian multisource data fusion, weighted linear combinations, and deep learning architectures capable of capturing complex multifactor interactions.

Fifth, the species occurrence data exhibited representational limitations. Despite applying data cleaning and spatial thinning to reduce sampling bias, insufficient sample sizes and uneven geographical coverage may compromise model generalizability, particularly at range margins or in undersampled desert regions [70]. Future studies should incorporate remote sensing products, citizen science data, and systematic field monitoring to improve the spatial completeness and temporal continuity of the occurrence records.

Sixth,the assumption of static soil conditions introduces uncertainty. Projecting soil variables as static under future scenarios, although common in ENM, fails to capture soil dynamics driven by climate change, land-use changes, and altered hydrological regimes. This assumption may underestimate the impact of environmental restructuring on the species distribution. Future projections can be enhanced by integrating soil-process models, scenario-based simulations, and high-resolution soil databases.

Finally, the present study used a correlative ENM framework, which, while interpretable and robust, does not yet incorporate explicit host–parasite interaction mechanisms. Future studies should aim to develop joint species distribution models (JSDMs) or coupled SDMs that capture co-occurrence dependencies and feedback processes within host-parasite systems. Mechanistic or process-based modeling approaches should also be explored to simulate key environmental drivers—such as soil moisture dynamics, hydrological fluxes, and host physiological responses —under changing climate conditions.

Despite certain limitations, this study established a novel methodological framework using dual-dimensional niche modeling and multifactor analysis. This study provides critical insights into the distributional responses and climatic vulnerability of keystone species in arid ecosystems. Future studies should prioritize cross-scale data integration, dynamic soil process modeling, and the advanced quantification of non-linear interactions. These advances will facilitate the development of ecologically realistic predictive modeling systems, thereby strengthening the scientific basis for desert ecosystem management, species conservation, and climate adaptation planning.

## Summary

This study systematically evaluated the potential distribution of *H. ammodendron* and *C. deserticola* under current and future climate scenarios using an integrated dual-model approach based on climatic and edaphic variables. The ensemble model demonstrated superior performance across all the evaluation metrics, confirming the reliability of the predictions.

Compared with single-model approaches, the strategy of independently constructing climatic and soil suitability models, followed by integration using the minimum suitability rule, effectively mitigated variable collinearity and prediction bias. Among the climatic factors, bio4, bio9, and bio15 had the most significant influence, particularly on *C. deserticola*, highlighting their sensitivity to climatic and dry season conditions. Key edaphic factors included available water capacity, soil texture, and soil type, with *C. deserticola* showing greater sensitivity to soil variability, reflecting its strong dependence on the rhizosphere microenvironment of its host plant.

Currently, both species are predominantly distributed in arid regions of northwestern China and southern Mongolia. Projections indicated a general reduction in suitable habitats under future climate scenarios for both species. However, *C. deserticola* is expected to experience a more pronounced range of contraction, indicating greater vulnerability to climate change. Although *C. deserticola* maintains a strong spatial overlap and persistent obligate dependence on its host, *H. ammodendron*, in future scenarios, the decreasing congruence between climatic and soil niche suitability for both species suggests that increasing environmental heterogeneity may disrupt ecological synchrony.

Consequently, future climate change poses a substantial threat to *H. ammodendron*–*C. deserticola* symbiotic system, presenting particularly acute challenges to the survival of the parasitic *C. deserticola*. The dual-model integration framework proposed in this study offers a robust scientific basis for predicting habitat suitability among complex interacting species and guiding strategic conservation planning in desert ecosystems.

## Supporting information

S1 File Supplemental Tables and Figures. (DOCX)
