# Supplementary material for: Dual niche modeling with GEE and SHAP for predicting habitat shifts of Haloxylon ammodendron and Cistanche deserticola under climate change
Source: PLoS One. 2025 Dec 19;20(12):e0338809. doi: 10.1371/journal.pone.0338809 (PMC12716759; doi:10.1371/journal.pone.0338809)
Supplement: S1 File — (DOCX) [file pone.0338809.s001.docx]

**Supplementary**

**Table S1** Initially selected environment variables.

| **Variables** | **Description** | **Unit** |
| --- | --- | --- |
| **bio1** | Annual Mean Temperature | ℃ |
| bio2 | Mean Diurnal Range | ℃ |
| bio3 | Isothermally | % |
| **bio4** | Temperature Seasonality | Standard Deviation ×100 |
| bio5 | Max Temperature of Warmest Month | ℃ |
| bio6 | Min Temperature of Coldest Month | ℃ |
| bio7 | Annual Range of Temperature | ℃ |
| **bio8** | Mean Temperature of Wettest Quarter | ℃ |
| **bio9** | Mean Temperature of Driest Quarter | ℃ |
| bio10 | Mean Temperature of Warmest Quarter | ℃ |
| bio11 | Mean Temperature of Coldest Quarter | ℃ |
| **bio12** | Annual Precipitation | mm |
| bio13 | Precipitation of Wettest Month | mm |
| bio14 | Precipitation of Driest Month | mm |
| **bio15** | Precipitation Seasonality | Coefficient of Variation |
| bio16 | Precipitation of Wettest Quarter | mm |
| bio17 | Precipitation of Driest Quarter | mm |
| bio18 | Mean Precipitation of Warmest Quarter | mm |
| bio19 | Mean Precipitation of Coldest Quarter | mm |
| **HWSD2_ID** | Unique identifier for the HWSD2 soil mapping unit | - (ID) |
| **WISE30s_ID** | Unique identifier for WISE30s | - (ID) |
| **COVERAGE** | Coverage area of the soil mapping unit | % |
| **SHARE** | Share of the soil unit in the grid cell | % |
| WRB4 | World Reference Base (WRB) classification | - |
| **WRB_PHASES** | WRB soil phase classification | - |
| **WRB2_CODE** | Secondary WRB classification | - |
| FAO90 | FAO 1990 soil classification | - |
| **KOPPEN** | Köppen climate classification | - |
| **TEXTURE_USDA** | Soil texture class (USDA classification) | - |
| **REF_BULK_DENSITY** | Reference bulk density of the soil | g/cm³ |
| **BULK_DENSITY** | Measured bulk density of the soil | g/cm³ |
| **DRAINAGE** | Soil drainage class (higher value = better drainage) | - |
| **ROOT_DEPTH** | Maximum rooting depth of soil | cm |
| **AWC** | Available water capacity of soil | mm/m |
| **PHASE1** | Additional phase information for soil unit | - |
| PHASE2 | Secondary additional phase information | - |
| ROOTS | Presence of roots in soil (indicator) | - |
| IL | Infiltration limitation indicator | - |
| ADD_PROP | Additional soil properties indicator | - |
| **elevation** | Elevation | m |
| **slope** | Slope | ° |
| **aspect** | Aspect | ° |
| hillshade | Hillshade | - |
| **HFP** | Human Footprint | - |
| **GDP** | Gross Domestic Product | USD |

**Note:** Variables highlighted in bold were retained for the final modeling stage.


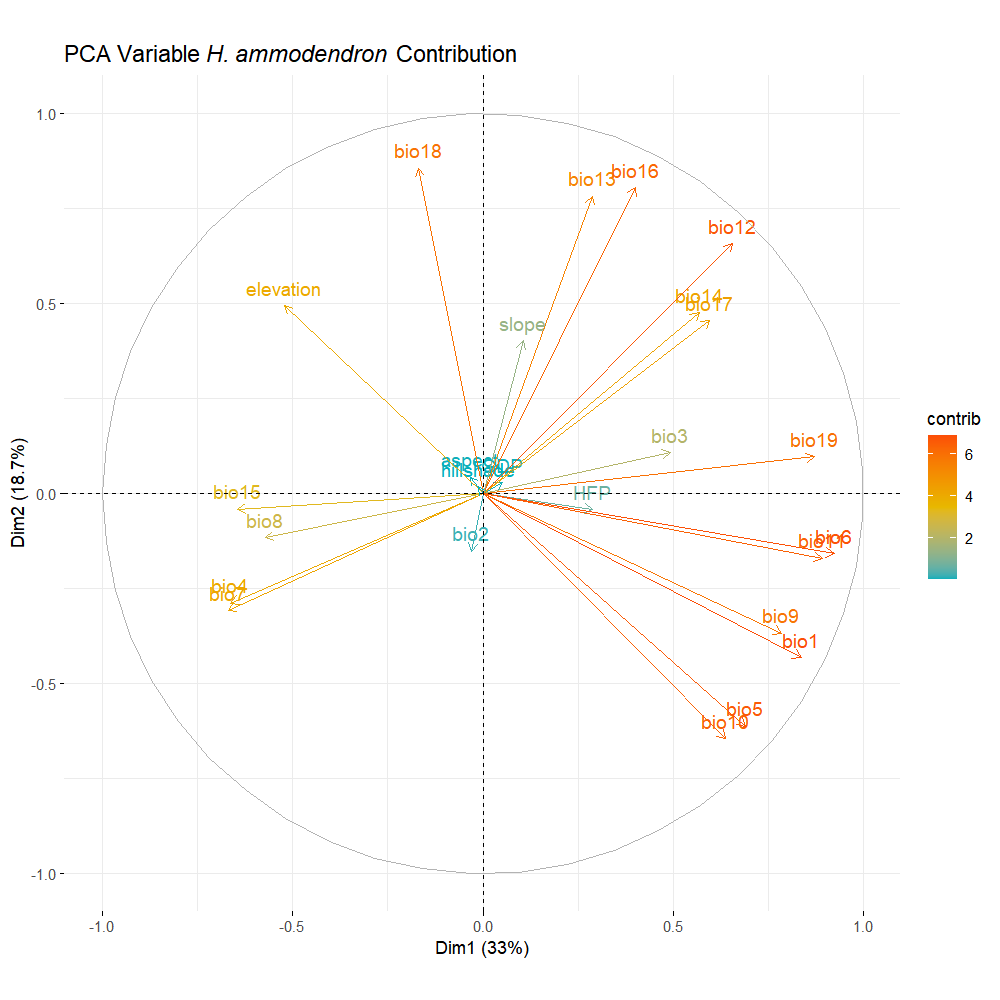

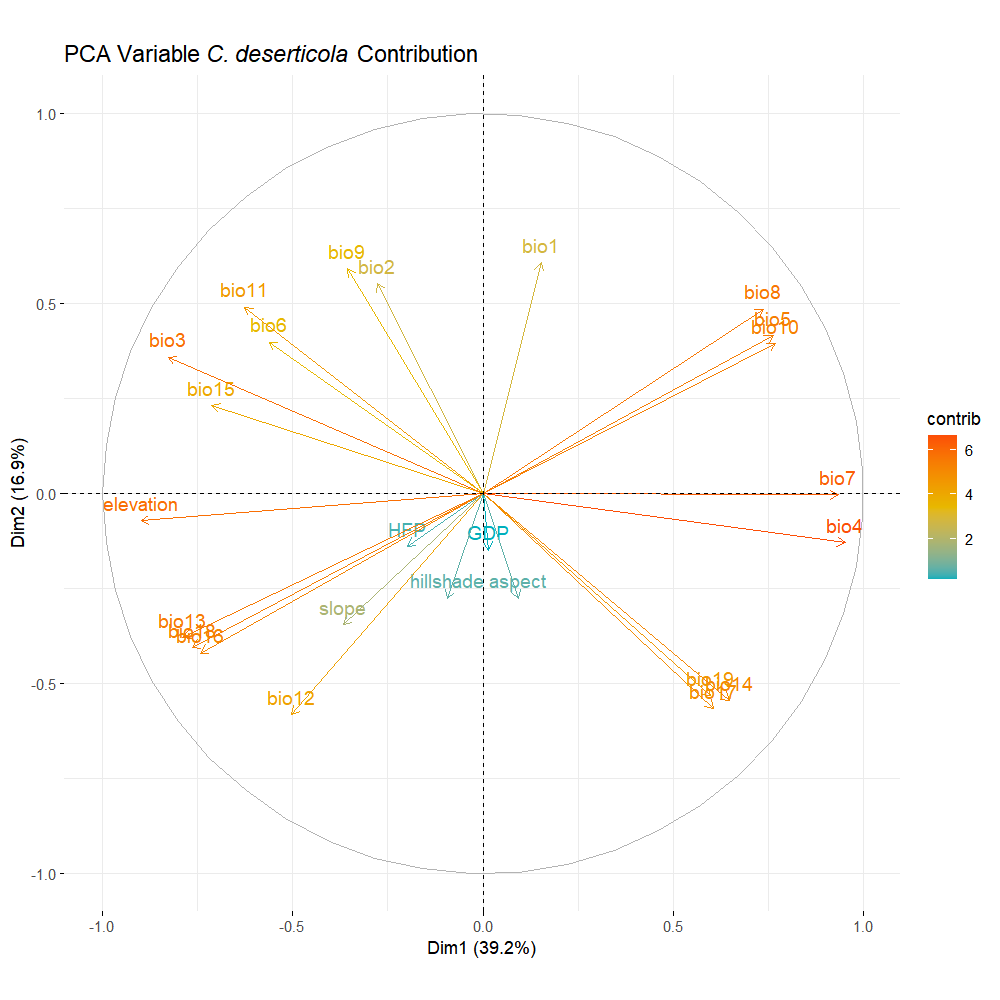


**Fig. S1.** Principal component analysis of environmental variables.


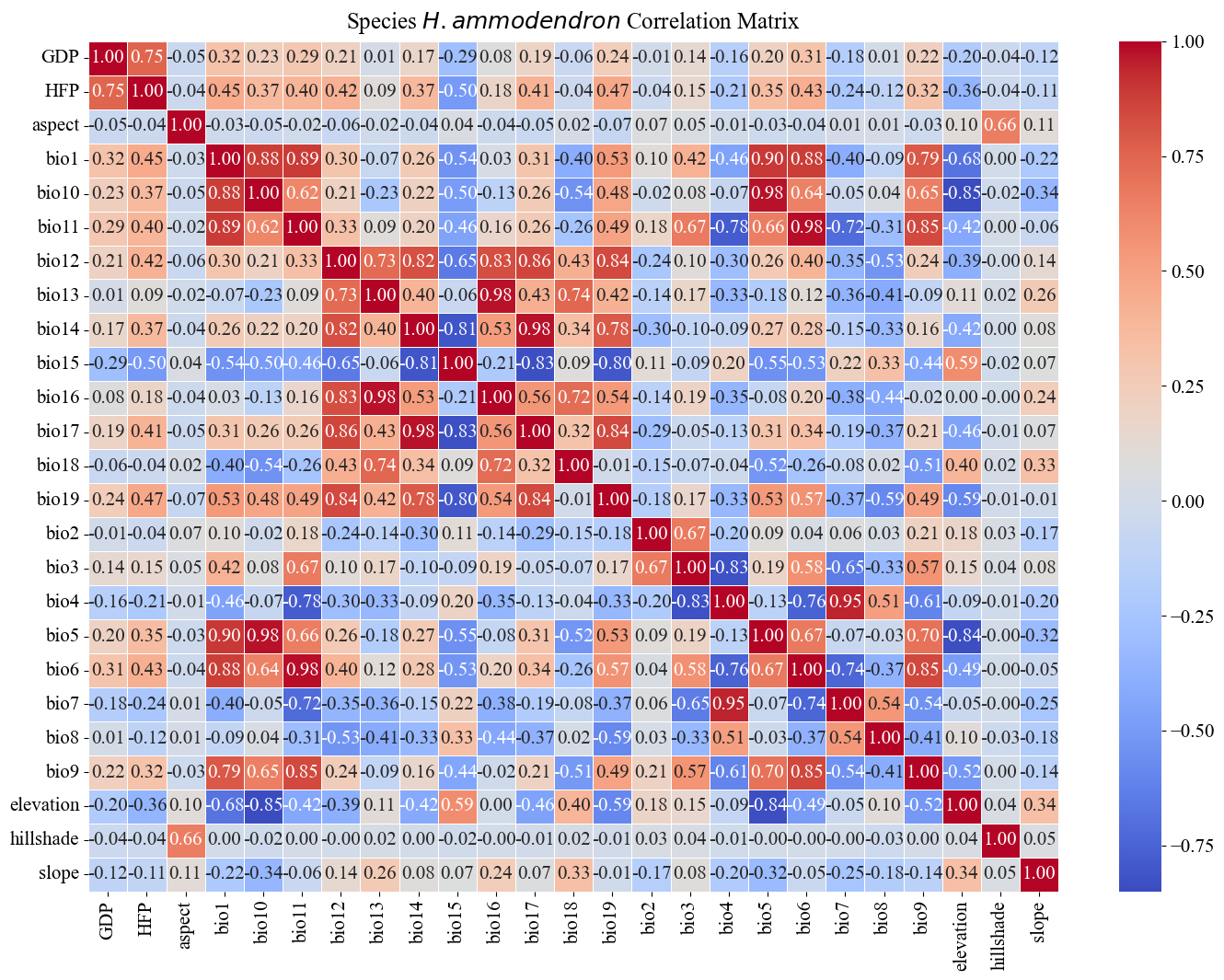

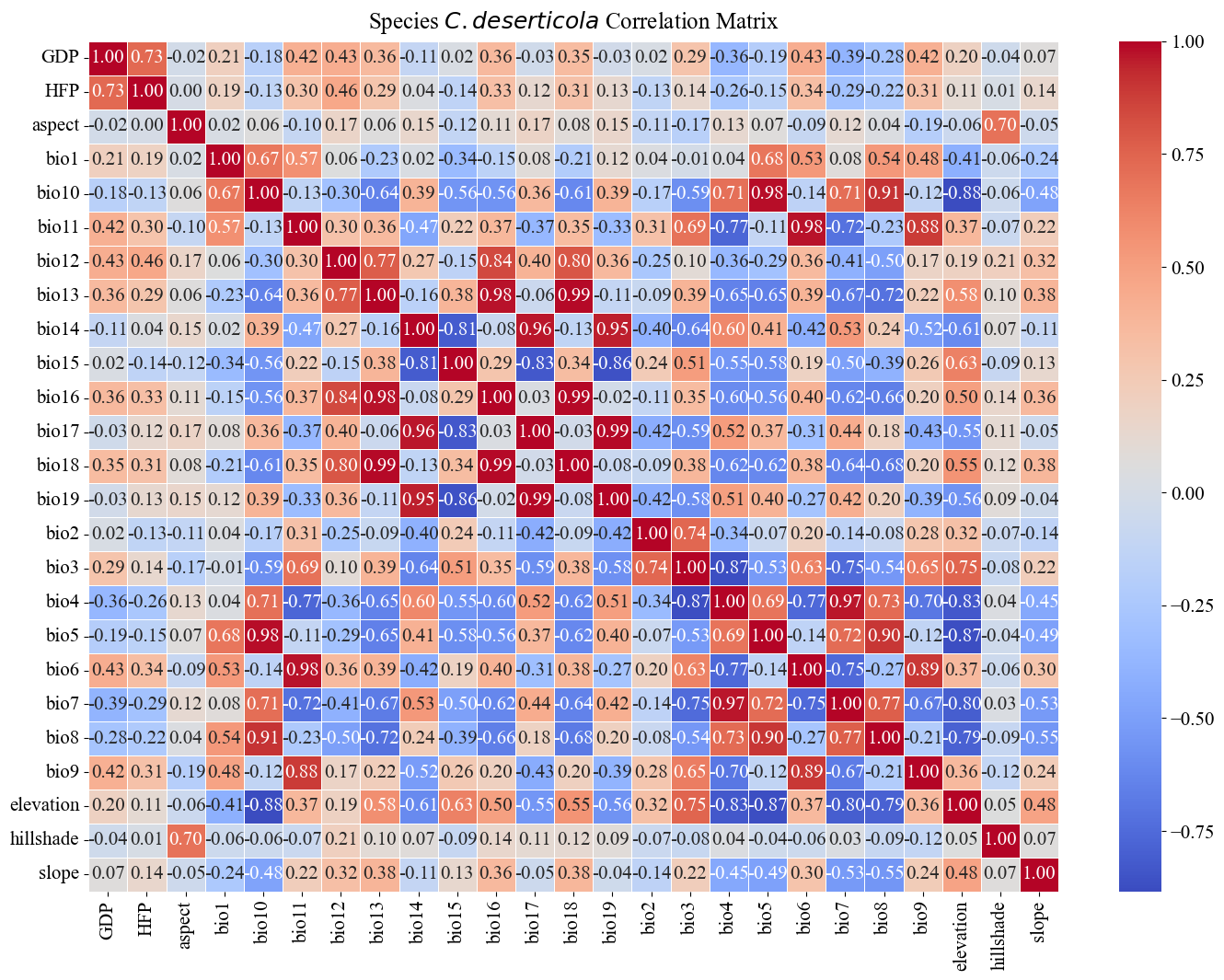


**Fig. S2.** Pearson correlation analysis of environmental variables.


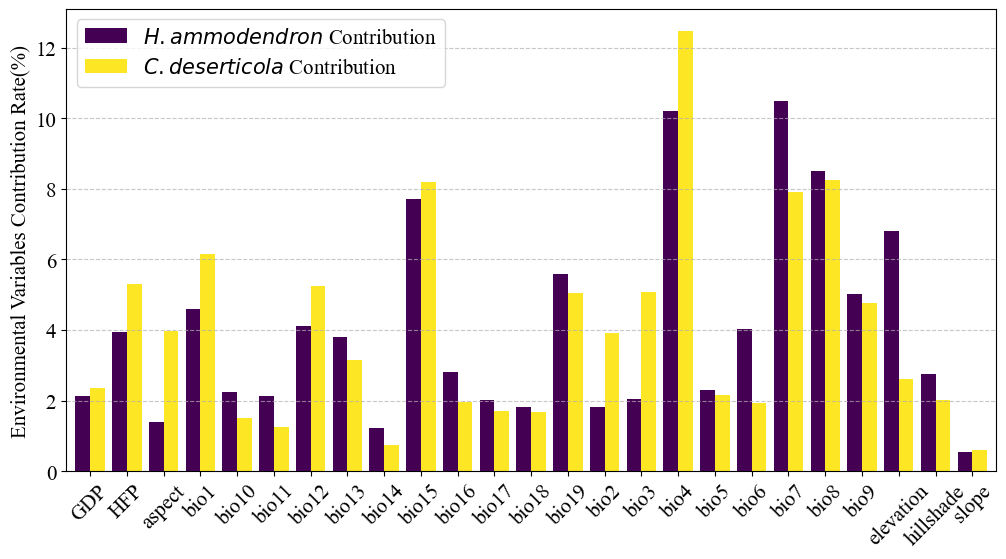


**Fig. S3.**The contribution rate of all environmental variables.


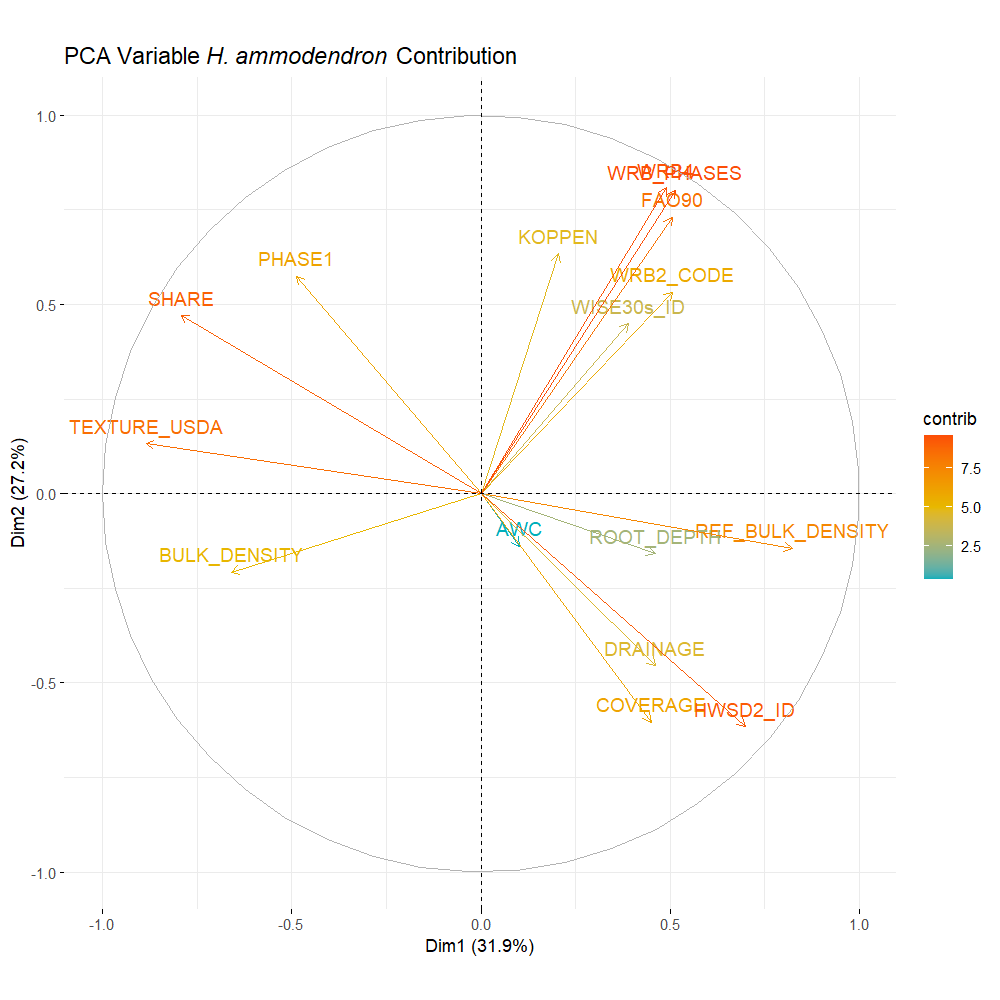

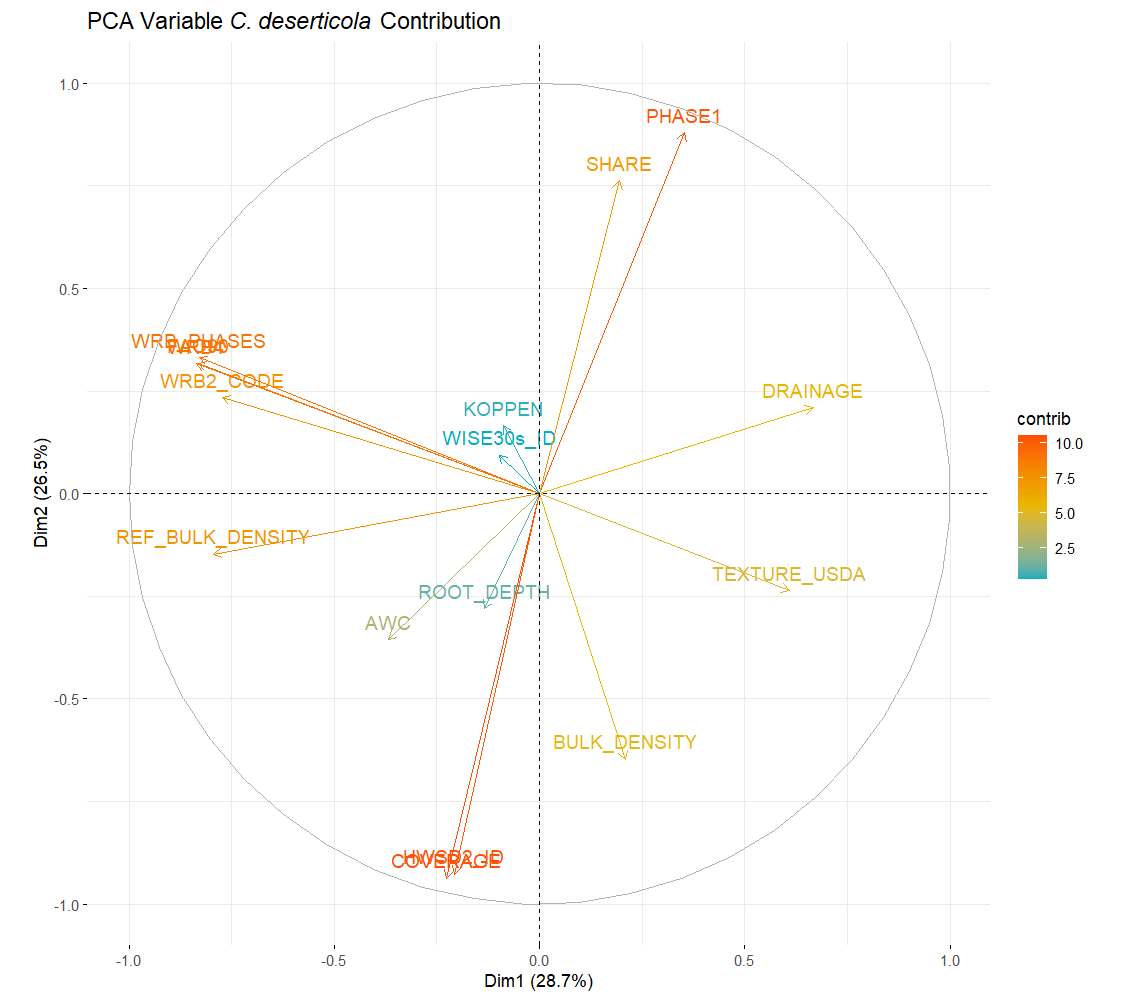


**Fig. S4.** Principal component analysis of soil variables.


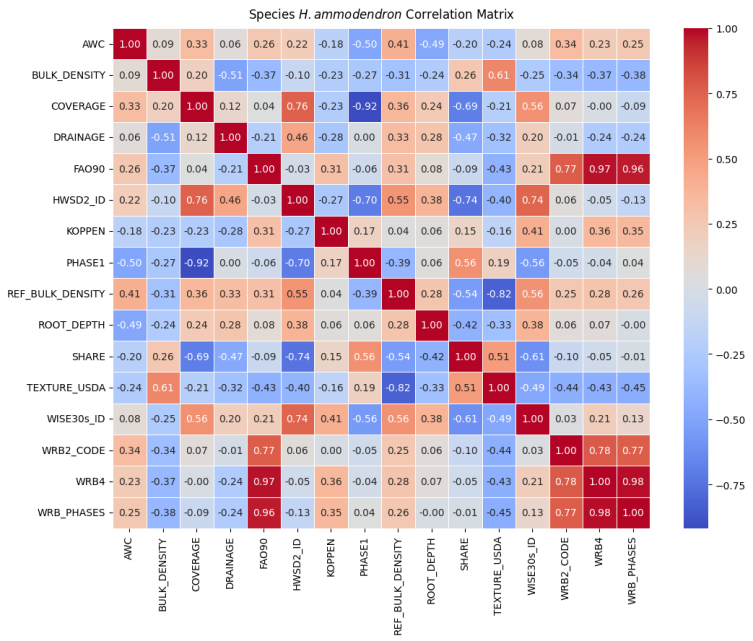


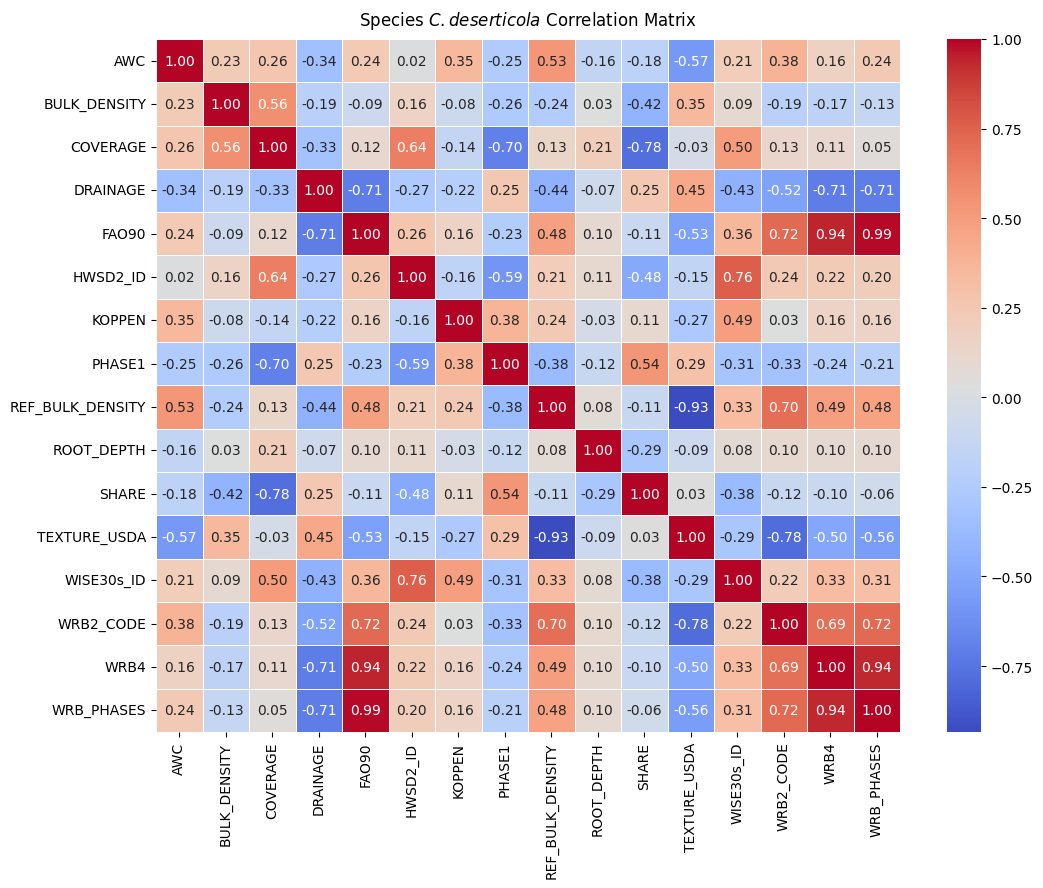


**Fig. S5.** Pearson correlation analysis of soil variables.


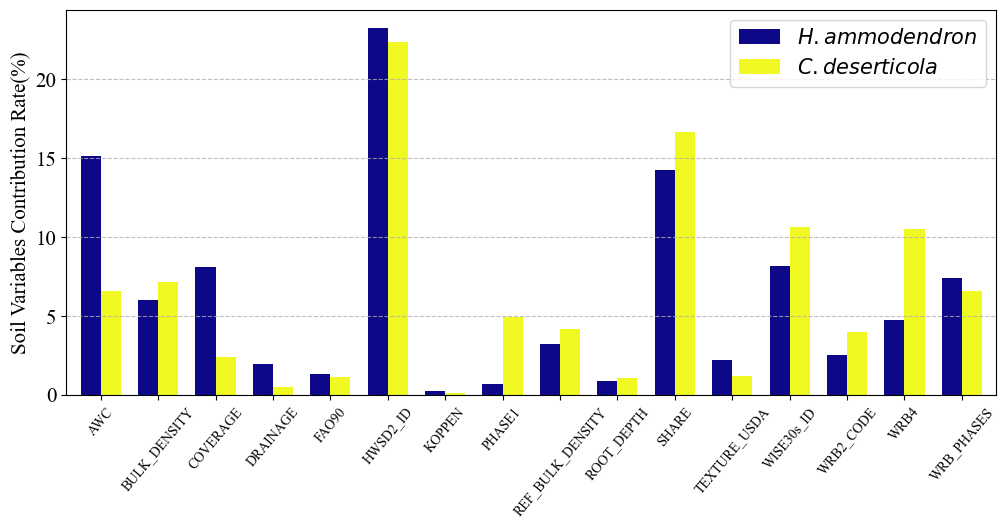


**Fig. S6.** The contribution rate of all soil variables.

*
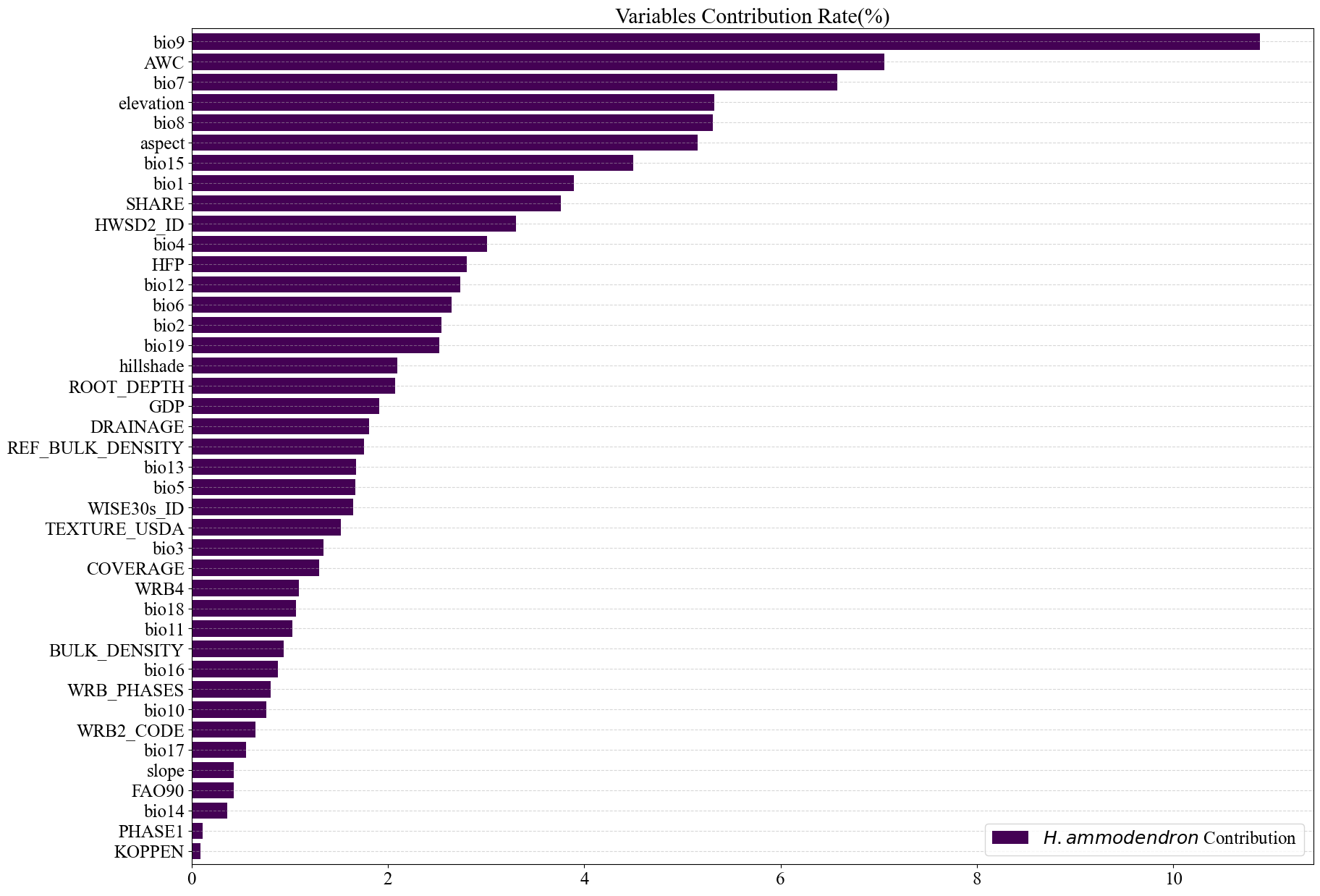
*

**Fig. S7.** The contribution rate of all variables.


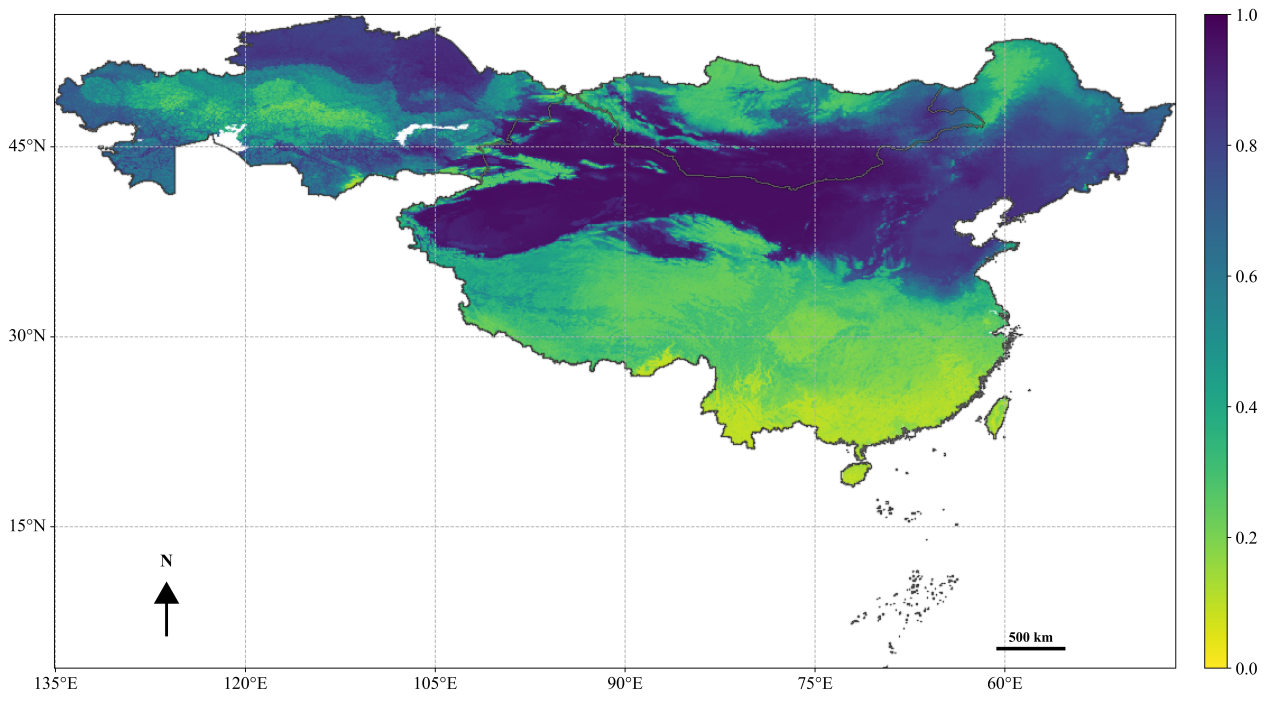


**Fig. S8.** Climate-Based Habitat Suitability Map for *H. ammodendron.*

Vector data were obtained from publicly available datasets in the GEE Data Catalog ([https://developers.google.com/earth-engine/datasets/catalog](https://developers.google.com/earth-engine/datasets/catalog)https://developers.google.com/earth-engine/datasets/catalog).


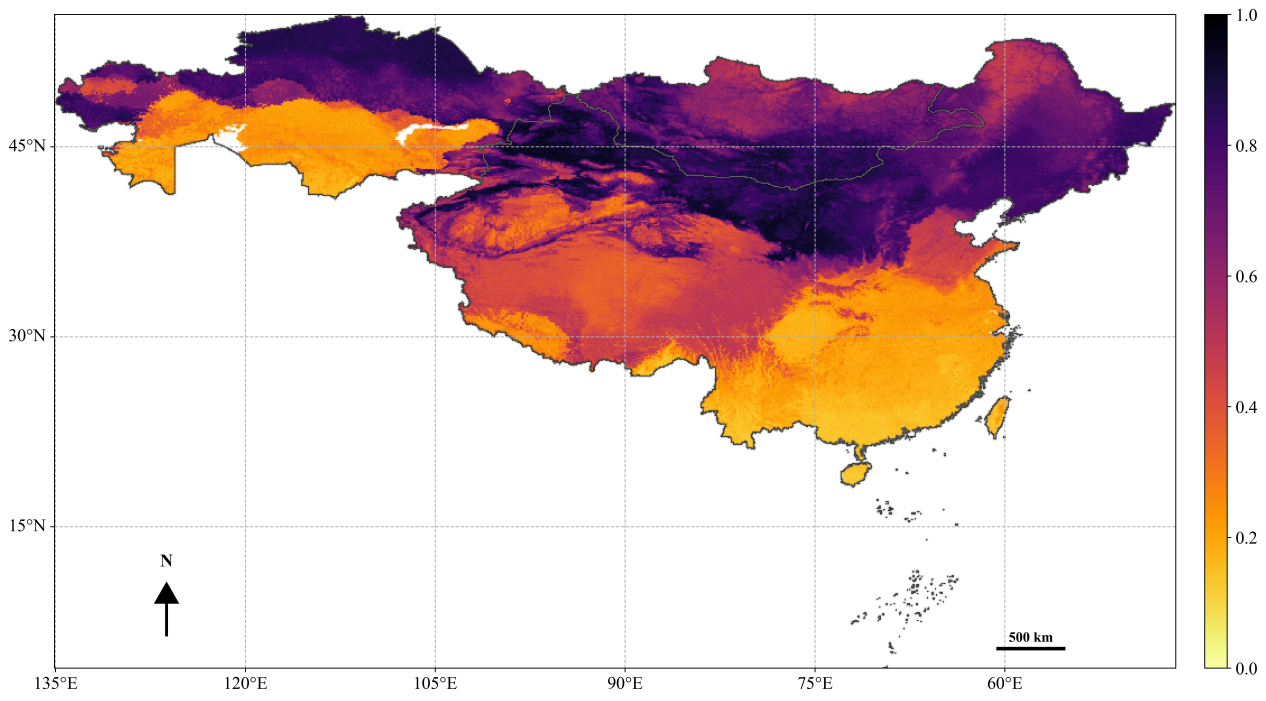


**Fig. S9.** Climate-Based Habitat Suitability Map for *C. deserticola.*

Vector data were obtained from publicly available datasets in the GEE Data Catalog ([https://developers.google.com/earth-engine/datasets/catalog](https://developers.google.com/earth-engine/datasets/catalog)https://developers.google.com/earth-engine/datasets/catalog).


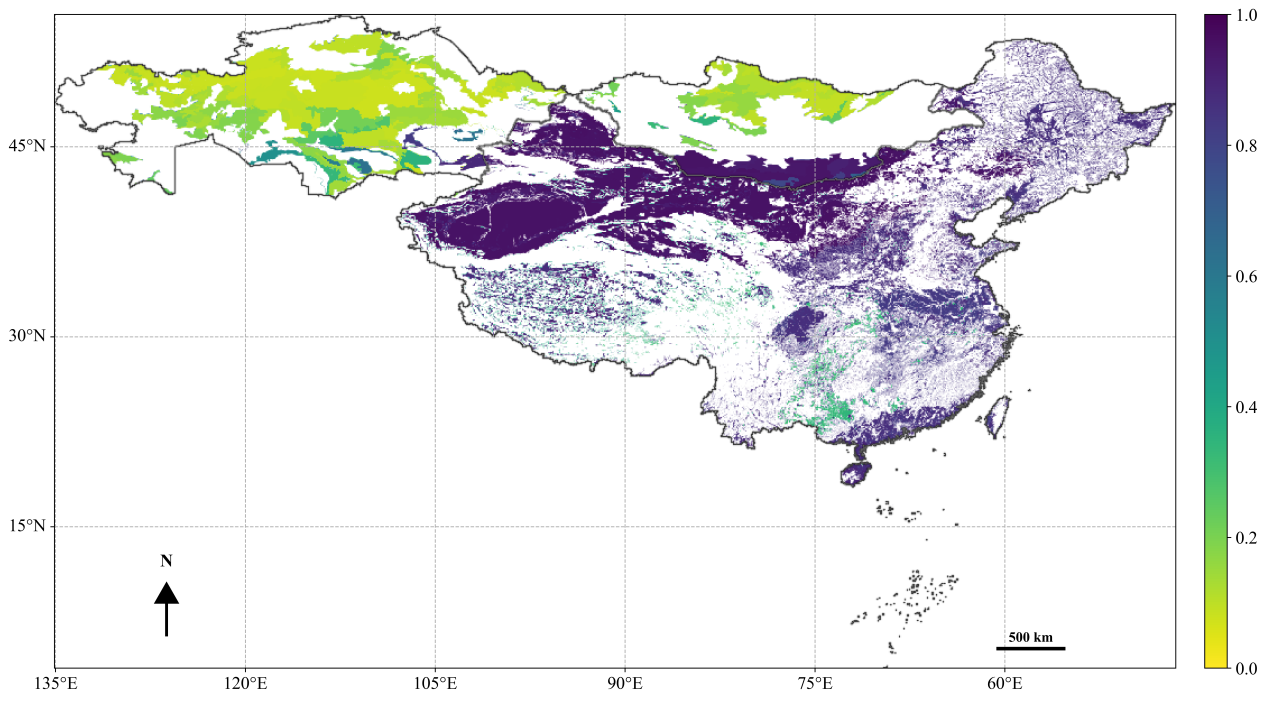


**Fig. S10.** Soil Adaptability Distribution of *H. ammodendron.*

Vector data were obtained from publicly available datasets in the GEE Data Catalog ([https://developers.google.com/earth-engine/datasets/catalog](https://developers.google.com/earth-engine/datasets/catalog)https://developers.google.com/earth-engine/datasets/catalog).

*
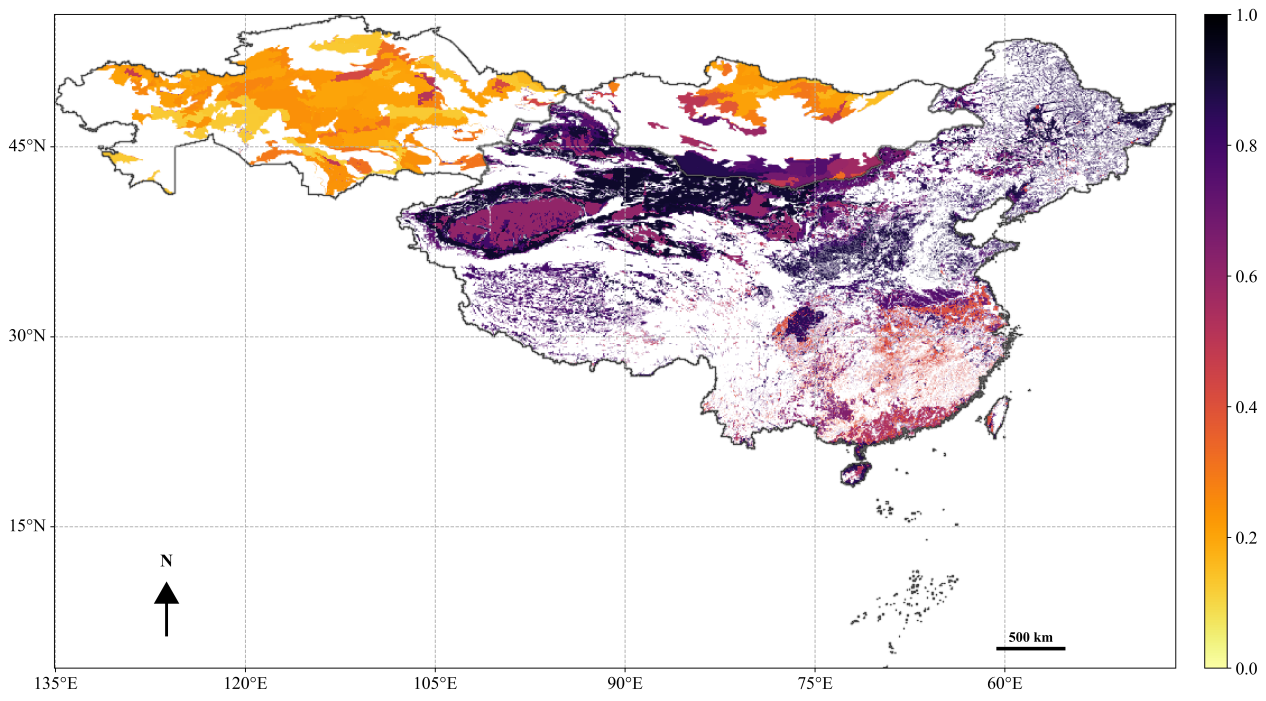
*

**Fig. S11.** Soil Adaptability Distribution of *C. deserticola.*

Vector data were obtained from publicly available datasets in the GEE Data Catalog ([https://developers.google.com/earth-engine/datasets/catalog](https://developers.google.com/earth-engine/datasets/catalog)https://developers.google.com/earth-engine/datasets/catalog).

**Table S2** Sensitivity analysis of Maximum Training Sensitivity and Specificity (MTSS) thresholds for *H. ammodendron* and *C. deserticola* (×10³km²).

| **Species** | **Scenario** | **Area ( MTSS_current)** | **Area**  **(MTSS × 0.95)** | **Area**  **(MTSS × 1.05)** |
| --- | --- | --- | --- | --- |
| ***H. ammodendron*** | Current | 2456.68 | 2520.12 | 2390.00 |
|  | SSP126 (2081–2100) | 1045.62 | 1071.00 | 1020.00 |
|  | SSP245 (2081–2100) | 1180.39 | 1208.00 | 1150.00 |
|  | SSP585 (2081–2100) | 1045.62 | 1071.00 | 1020.00 |
| ***C. deserticola*** | Current | 1258.01 | 1275.00 | 1240.00 |
|  | SSP126 (2081–2100) | 35.79 | 37.50 | 34.00 |
|  | SSP245 (2081–2100) | 25.78 | 27.00 | 24.50 |
|  | SSP585 (2081–2100) | 24.02 | 25.00 | 23.50 |

**Table S3** The metrics of *H. ammodendron* were assessed through 10 climatic niche model evaluations, and the following are the results for each evaluation.

| **runs** | **AUC-ROC** | **TSS** | **Boyce Index** | **Sensitivity** | **Specificity** | **Precision** |
| --- | --- | --- | --- | --- | --- | --- |
| 1 | 0.9562 | 0.8149 | 0.7796 | 0.9286 | 0.8295 | 0.8585 |
| 2 | 0.9525 | 0.7876 | 0.8689 | 0.9669 | 0.8000 | 0.8417 |
| 3 | 0.9479 | 0.8389 | 0.8969 | 0.9448 | 0.8469 | 0.9013 |
| 4 | 0.9693 | 0.8692 | 0.7966 | 0.9533 | 0.8692 | 0.8793 |
| 5 | 0.9393 | 0.7785 | 0.8958 | 0.9660 | 0.7027 | 0.8114 |
| 6 | 0.9663 | 0.8150 | 0.9108 | 0.9800 | 0.7297 | 0.8305 |
| 7 | 0.9534 | 0.8404 | 0.8558 | 0.9421 | 0.8750 | 0.8976 |
| 8 | 0.9538 | 0.8229 | 0.8609 | 0.9688 | 0.7604 | 0.8017 |
| 9 | 0.9635 | 0.8356 | 0.8720 | 0.9793 | 0.7638 | 0.8256 |
| 10 | 0.9572 | 0.7652 | 0.8377 | 0.9041 | 0.8396 | 0.8859 |

**Table S4** The metrics of *C. deserticola* were assessed through 10 climatic niche model evaluations, and the following are the results for each evaluation.

| **runs** | **AUC-ROC** | **TSS** | **Boyce Index** | **Sensitivity** | **Specificity** | **Precision** |
| --- | --- | --- | --- | --- | --- | --- |
| 1 | 0.9161 | 0.7688 | 0.9689 | 1 | 0.7333 | 0.8889 |
| 2 | 0.9086 | 0.7402 | 0.9222 | 0.9855 | 0.6923 | 0.85 |
| 3 | 0.8930 | 0.6602 | 0.9301 | 0.9574 | 0.6061 | 0.7759 |
| 4 | 0.9205 | 0.7839 | 0.8892 | 0.9348 | 0.6857 | 0.7963 |
| 5 | 0.9492 | 0.8438 | 0.8737 | 1 | 0.7813 | 0.8205 |
| 6 | 0.9417 | 0.8172 | 0.815 | 0.9643 | 0.8529 | 0.9153 |
| 7 | 0.9186 | 0.7066 | 0.9444 | 0.907 | 0.7308 | 0.8478 |
| 8 | 0.9502 | 0.8031 | 0.8667 | 0.9552 | 0.8095 | 0.8889 |
| 9 | 0.9459 | 0.7749 | 0.9553 | 0.9655 | 0.7500 | 0.8615 |
| 10 | 0.8551 | 0.6978 | 0.9777 | 0.9778 | 0.600 | 0.8148 |

**Table S5** The metrics of *H. ammodendron* were assessed through 10 soil niche model evaluations, and the following are the results for each evaluation.

| **runs** | **AUC-ROC** | **TSS** | **Sensitivity** | **Specificity** | **Precision** |
| --- | --- | --- | --- | --- | --- |
| 1 | 0.9669 | 0.8718 | 0.9286 | 0.9432 | 0.8864 |
| 2 | 0.9725 | 0.8973 | 0.9245 | 0.9545 | 0.9074 |
| 3 | 0.9619 | 0.8757 | 0.9455 | 0.8660 | 0.8000 |
| 4 | 0.9879 | 0.9472 | 0.9565 | 0.9626 | 0.9167 |
| 5 | 0.9684 | 0.9173 | 0.9744 | 0.9292 | 0.9048 |
| 6 | 0.9829 | 0.9265 | 0.9516 | 0.9640 | 0.9365 |
| 7 | 0.9673 | 0.8997 | 0.9474 | 0.9333 | 0.8852 |
| 8 | 0.9575 | 0.8558 | 0.8974 | 0.9479 | 0.8750 |
| 9 | 0.9741 | 0.9294 | 0.9688 | 0.9449 | 0.8986 |
| 10 | 0.9650 | 0.9066 | 0.9153 | 0.9655 | 0.9310 |

**Table S6** The metrics of *C. deserticola* were assessed through 10 soil niche model evaluations, and the following are the results for each evaluation.

| **runs** | **AUC-ROC** | **TSS** | **Sensitivity** | **Specificity** | **Precision** |
| --- | --- | --- | --- | --- | --- |
| 1 | 0.9261 | 0.9716 | 0.9783 | 0.7000 | 0.8333 |
| 2 | 0.9163 | 0.6410 | 1.0000 | 0.5897 | 0.6923 |
| 3 | 0.8831 | 0.6857 | 1.0000 | 0.6571 | 0.7600 |
| 4 | 0.9441 | 0.7815 | 0.9565 | 0.8286 | 0.7857 |
| 5 | 0.9261 | 0.7188 | 1.0000 | 0.7188 | 0.7097 |
| 6 | 0.9475 | 0.7647 | 1.0000 | 0.7647 | 0.7778 |
| 7 | 0.9108 | 0.6958 | 0.9091 | 0.6923 | 0.7143 |
| 8 | 0.9515 | 0.8095 | 0.8205 | 0.8571 | 0.8421 |
| 9 | 0.8746 | 0.6136 | 0.9697 | 0.6111 | 0.6957 |
| 10 | 0.8775 | 0.6002 | 0.7778 | 0.6765 | 0.6563 |

**Table S7** Average Evaluation Metrics of the Climatic Niche Model.

| **Species** | **Model** | **AUC-ROC** | **TSS** | **Boyce Index** | **Sensitivity** | **Specificity** | **Precision** |
| --- | --- | --- | --- | --- | --- | --- | --- |
| *H. ammodendron* | RF_simple | 0.9427±0.0180 | 0.7677±0.0584 | 0.8583±0.0397 | 0.9588±0.0273 | 0.7052±0.0645 | 0.7965±0.0389 |
| *C. deserticola* |  | 0.8905±0.0411 | 0.7251±0.0712 | 0.9673±0.0217 | 0.9543± 0.0302 | 0.6205±0.1116 | 0.7995±0.0594 |
| *H. ammodendron* | RF_interm | 0.9544±0.0125 | 0.8118±0.0408 | 0.8374±0.0631 | 0.9502±0.0253 | 0.7933±0.0693 | 0.8486±0.0417 |
| *C. deserticola* |  | 0.9158±0.0331 | 0.7526±0.0695 | 0.9101±0.0511 | 0.9589±0.0329 | 0.7094±0.0927 | 0.8389±0.0485 |
| *H. ammodendron* | GBDT_simple | 0.9539±0.0094 | 0.8018±0.0339 | 0.8853±0.0381 | 0.9496±0.0166 | 0.7903±0.0561 | 0.8452±0.0389 |
| *C. deserticola* |  | 0.9174±0.0368 | 0.7465±0.0732 | 0.8904±0.0655 | 0.9648±0.0288 | 0.7265±0.0826 | 0.8475±0.0434 |
| *H. ammodendron* | GBDT_interm | 0.9562±0.0050 | 0.8168±0.0261 | 0.8326±0.0522 | 0.9464±0.0286 | 0.8352±0.0446 | 0.8736±0.0300 |
| *C. deserticola* |  | 0.9216±0.0323 | 0.7511±0.0626 | 0.8936±0.0522 | 0.9407±0.0335 | 0.7545±0.0861 | 0.8592±0.0418 |
| *H. ammodendron* | Ensemble Model | 0.9559 ± 0.0089 | 0.8168 ± 0.0319 | 0.8575 ± 0.0426 | 0.9534 ± 0.0240 | 0.8017 ± 0.0599 | 0.8534 ± 0.0363 |
| *C. deserticola* |  | 0.9199 ± 0.0298 | 0.7596 ± 0.0578 | 0.9143 ± 0.0520 | 0.9648 ± 0.0288 | 0.7242 ± 0.0444 | 0.8460 ± 0.0806 |

**Table S8** Average Evaluation Metrics of the Soil Niche Model.

| **Species** | **Model** | **AUC-ROC** | **TSS** | **Sensitivity** | **Specificity** | **Precision** |
| --- | --- | --- | --- | --- | --- | --- |
| *H. ammodendron* | RF_simple | 0.9687±0.0107 | 0.8973±0.0251 | 0.9459±0.0291 | 0.9052±0.0319 | 0.8427±0.0578 |
| *C. deserticola* |  | 0.8863±0.0532 | 0.6695±0.0944 | 0.9665±0.0658 | 0.6075±0.1044 | 0.6998±0.0665 |
| *H. ammodendron* | RF_interm | 0.9715±0.0107 | 0.9083±0.0288 | 0.9437±0.0271 | 0.9423±0.0171 | 0.8977±0.0330 |
| *C. deserticola* |  | 0.9038±0.0517 | 0.7034±0.0827 | 0.9753±0.0564 | 0.6581±0.0954 | 0.7308±0.0578 |
| *H. ammodendron* | GBDT_simple | 0.9769±0.0086 | 0.9023±0.0297 | 0.9374±0.0273 | 0.9424±0.0126 | 0.8979±0.0227 |
| *C. deserticola* |  | 0.9205±0.0312 | 0.7124±0.0696 | 0.9409±0.0924 | 0.6992±0.1008 | 0.7495±0.0699 |
| *H. ammodendron* | GBDT_interm | 0.9723±0.0087 | 0.8950±0.0278 | 0.9374±0.0273 | 0.9437±0.0196 | 0.9015±0.0291 |
| *C. deserticola* |  | 0.9202±0.0320 | 0.7163±0.0718 | 0.8661±0.0912 | 0.7424±0.0924 | 0.7628±0.0674 |
| *H. ammodendron* | Ensemble Model | 0.9704 ± 0.0093 | 0.9027 ± 0.0287 | 0.9410 ± 0.0242 | 0.9411 ± 0.0292 | 0.8942 ± 0.0385 |
| *C. deserticola* |  | 0.9158 ± 0.0290 | 0.7026 ± 0.0707 | 0.9412 ± 0.0807 | 0.7096 ± 0.0865 | 0.7467 ± 0.0628 |


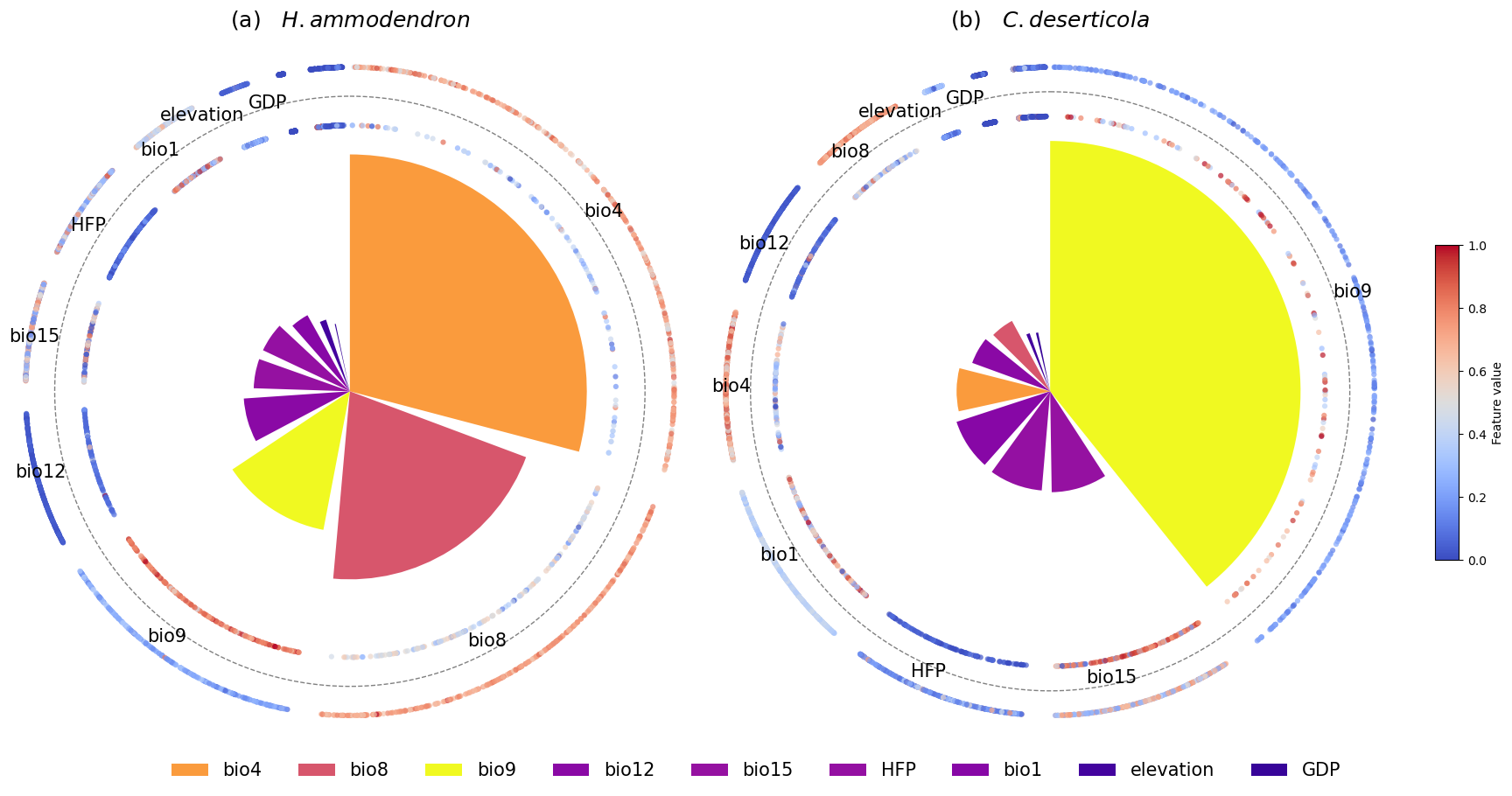


**Fig. S12.** Relative importance and spatial distribution patterns of environmental variables in ENMs for *H. ammodendron* and *C. deserticola.*


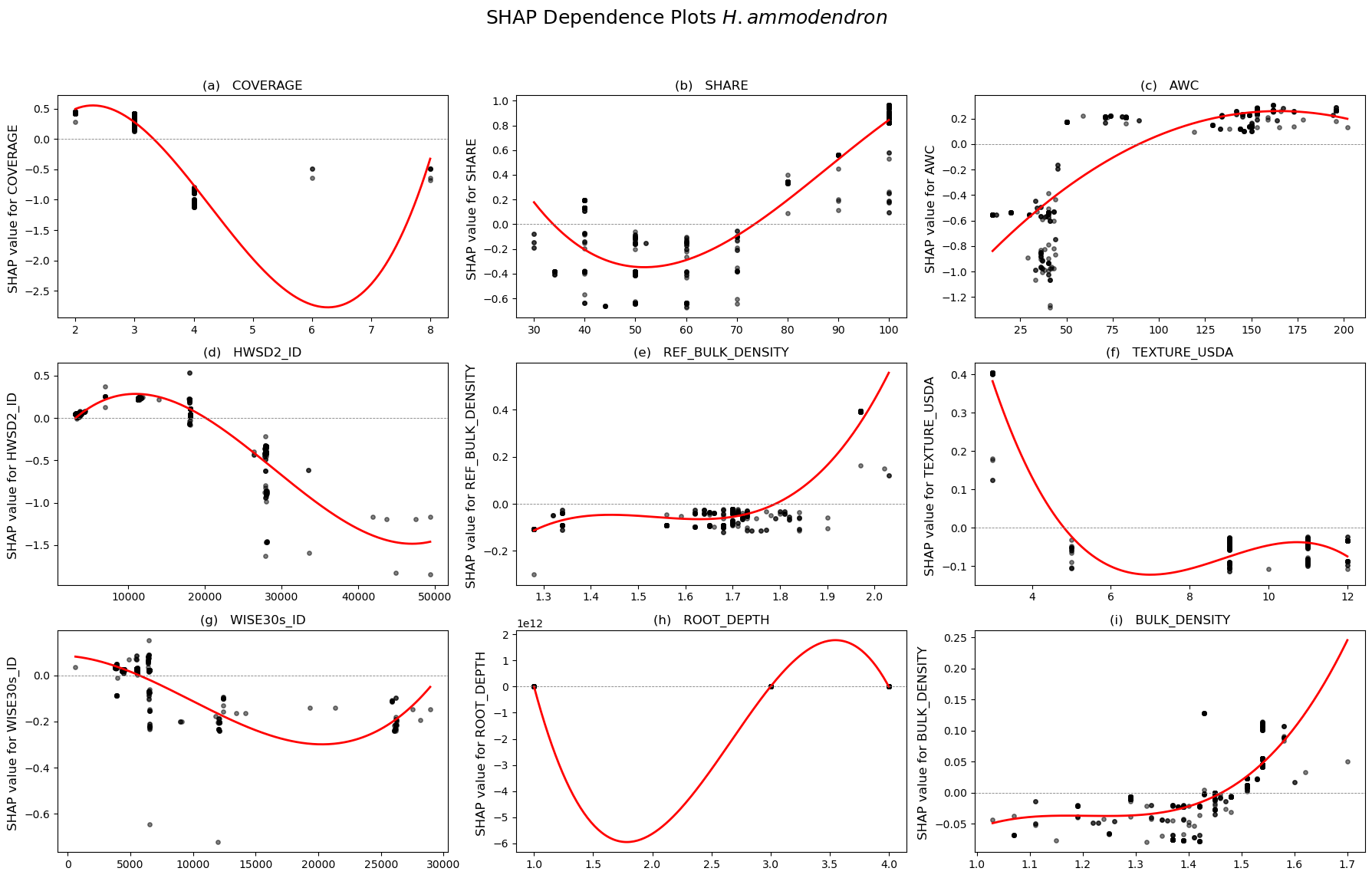


**Fig. S13.** SHAP Dependence Plot for *H. ammodendron.*


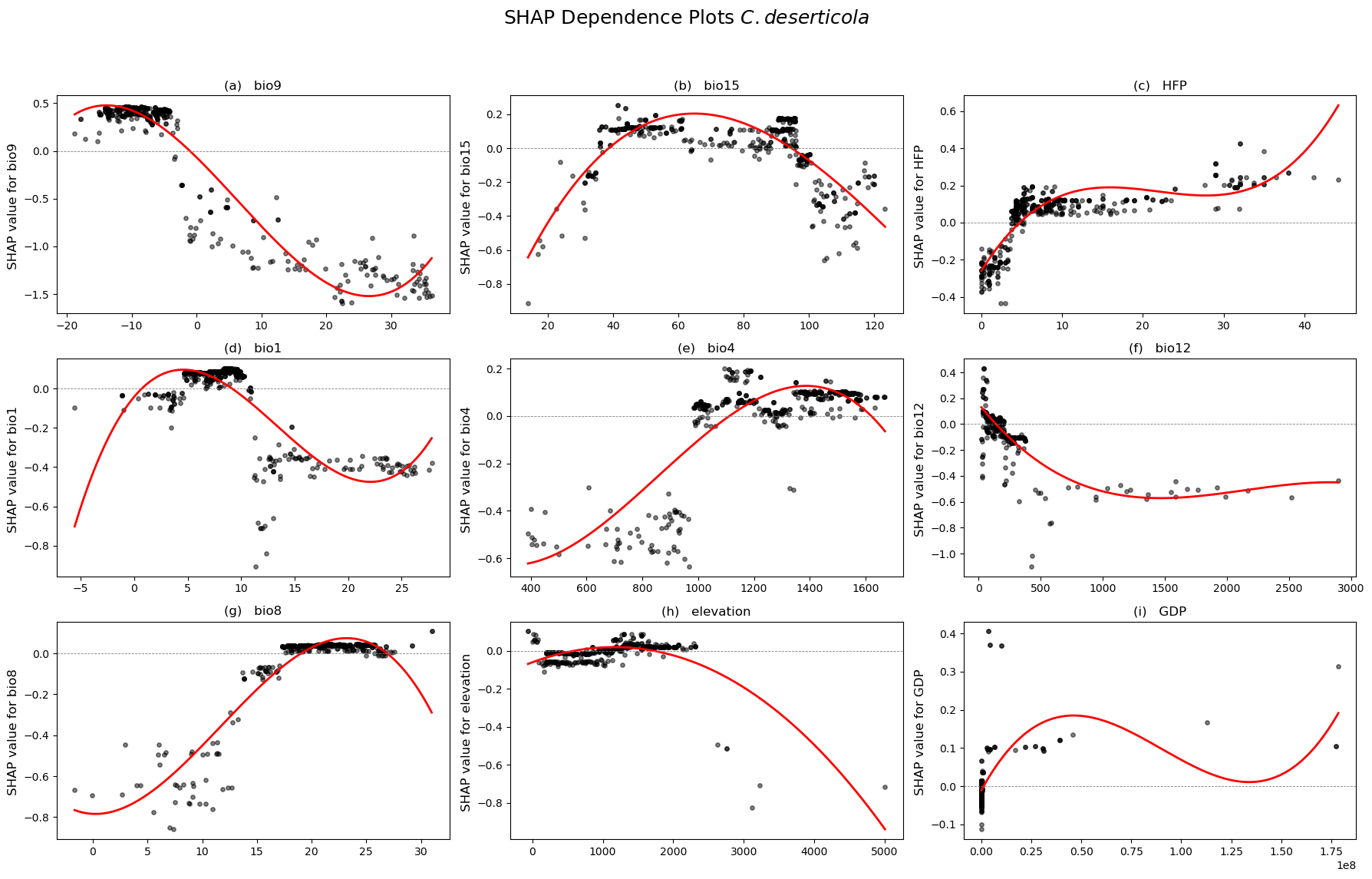
**Fig. S14.** SHAP Dependence Plot for *C. deserticola.*

*
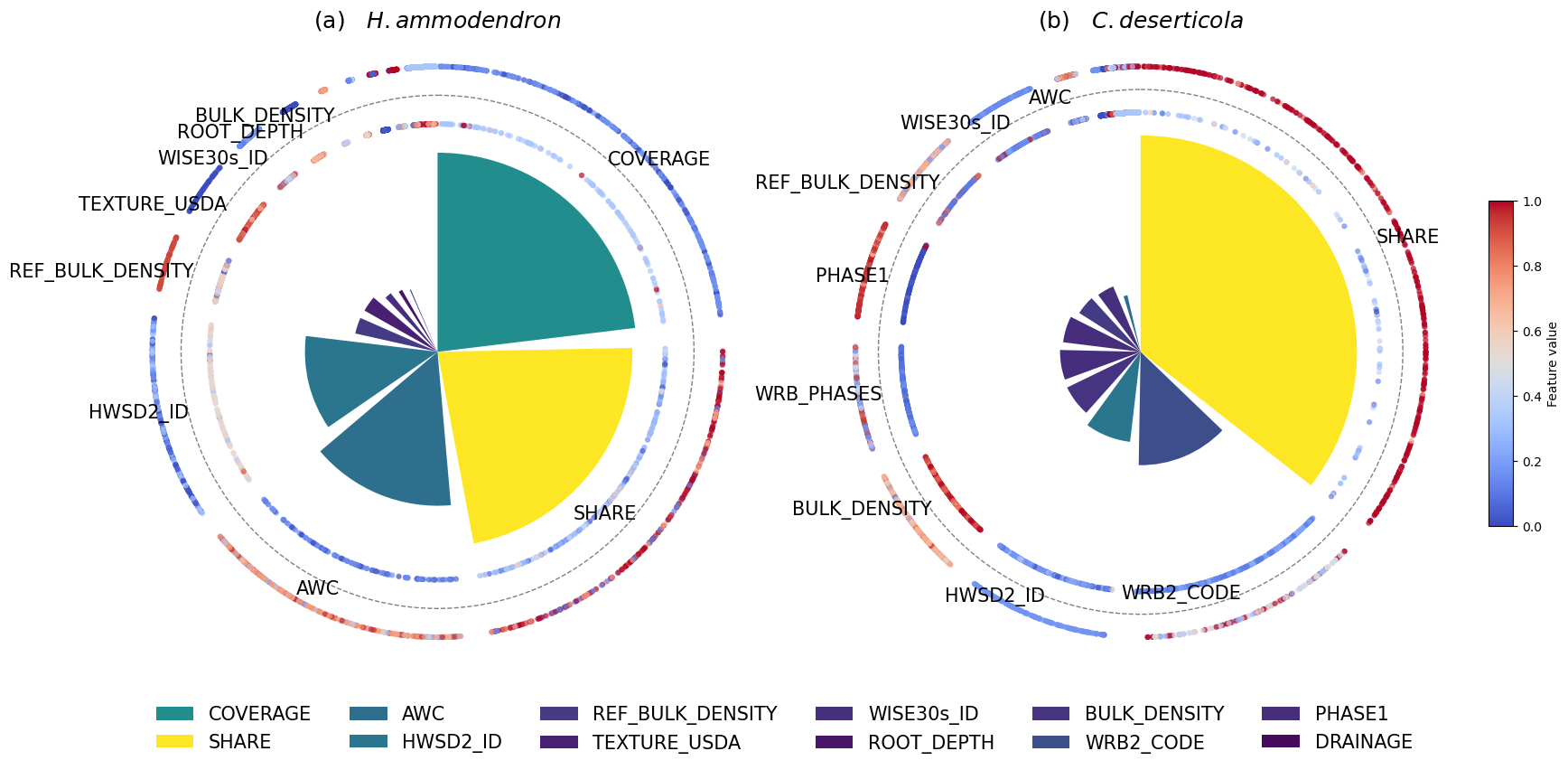
*

**Fig. S15.** Relative importance and spatial distribution patterns of soil variables in ENMs for *H. ammodendron* and *C. deserticola.*

*
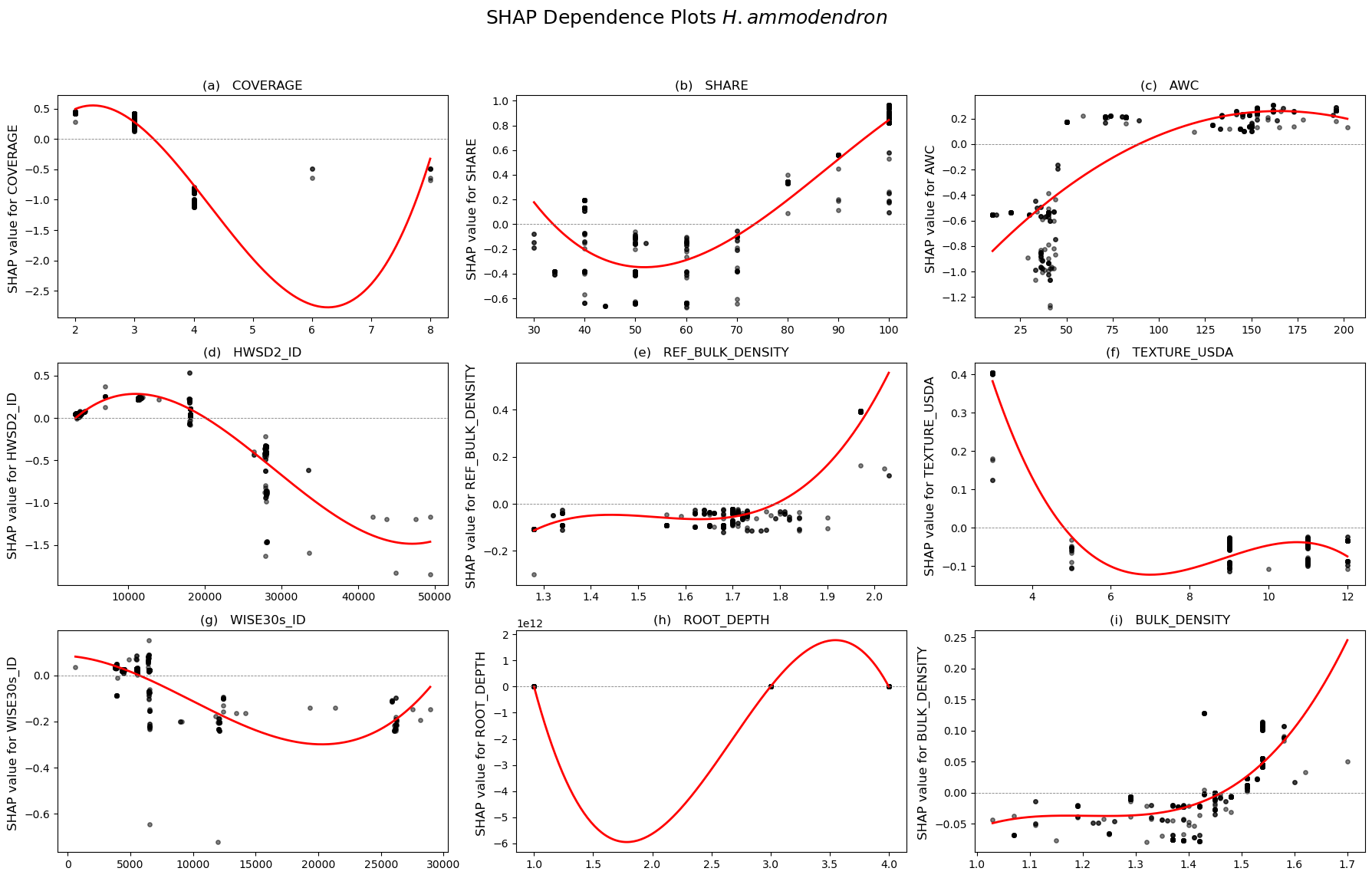
*

**Fig. S16.** SHAP Dependence Plot for *H. ammodendron.*

*
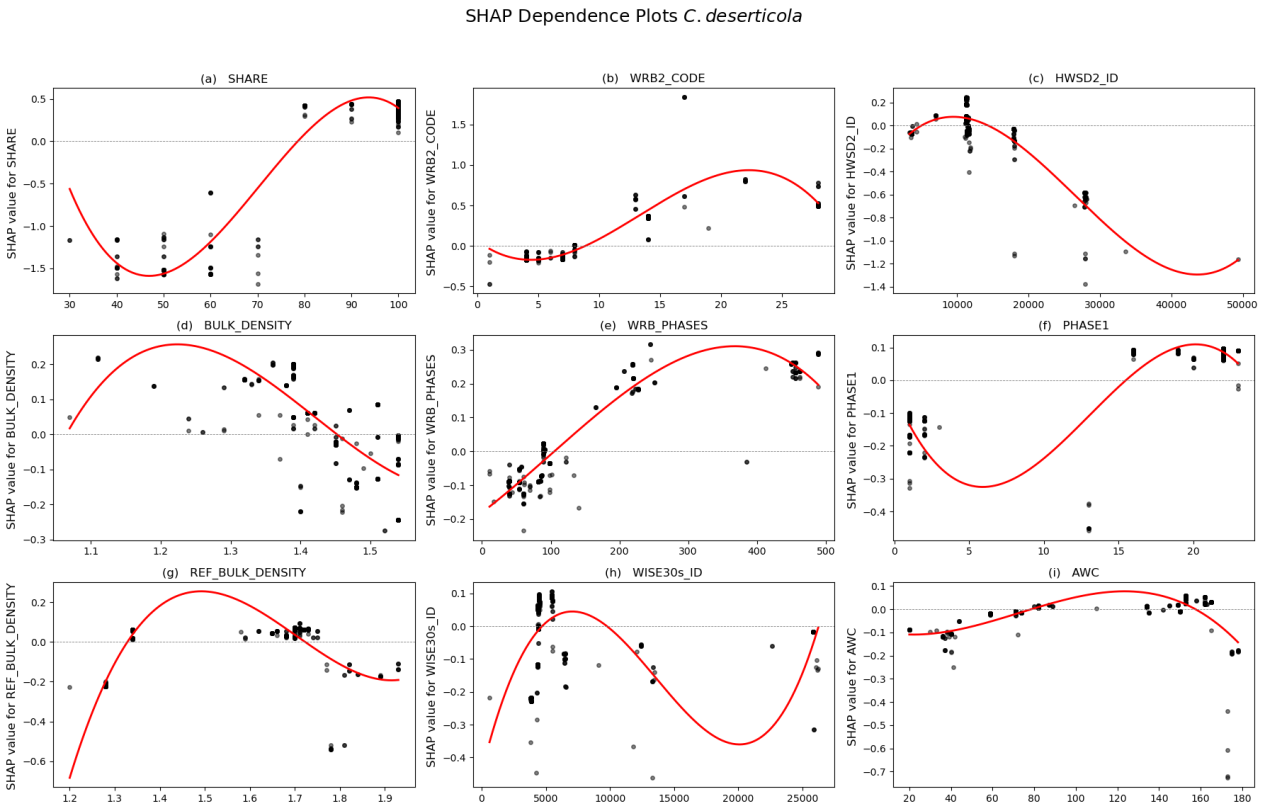
*

**Fig. S17.** SHAP Dependence Plot for *C. deserticola.*
